# Safety and Metabolism-Related Outcomes of Oral Nicotinamide Mononucleotide Supplementation in Adults: A Systematic Review and Meta-Analysis

**DOI:** 10.3390/nu18142251

**Published:** 2026-07-10

**Authors:** Wenyu Yang, Jun Huang, Zihan Tang, Cong Chen, Yanan Sun

**Affiliations:** 1 Medical Experimental Center, China Academy of Chinese Medical Sciences, Beijing 100700, China; yangwenyu202502@163.com (W.Y.); huangjun2001@foxmail.com (J.H.); tangzihan0629@163.com (Z.T.); 2 Department of Cardiology, Guang’anmen Hospital, China Academy of Chinese Medical Sciences, Beijing 100053, China

**Keywords:** nicotinamide mononucleotide, NMN, NAD+, dietary supplement, safety, adverse events, metabolic health, meta-analysis

## Abstract

**Background/Objectives**: Nicotinamide mononucleotide (NMN), a precursor of nicotinamide adenine dinucleotide (NAD^+^), is used as a dietary supplement, but its safety and metabolic effects in adults remain unclear. This review assessed the short-term safety and tolerability of oral NMN or NMN-related supplementation and examined metabolic and vascular outcomes. **Methods**: PubMed, Embase, Scopus, Web of Science, CNKI, and Wanfang were searched from inception to 13 May 2026. Eligible studies were parallel randomized controlled trials comparing oral NMN or NMN-related preparations with placebo, blank control, lifestyle control, or the same background intervention without NMN. Safety outcomes included adverse events, serious adverse events, withdrawals due to adverse events, system-specific adverse events, alanine aminotransferase, and aspartate aminotransferase. Random-effects models were used, with GRADE for evidence certainty. **Results**: Fifteen trials were included, with 10 contributing to safety analyses. NMN doses ranged from 250–2000 mg/day, and durations ranged from 14 days to 24 weeks. NMN did not increase overall, serious, withdrawal-related, or system-specific adverse events, nor did it significantly elevate ALT or AST. No significant effects were observed on body weight, BMI, fasting glucose, HbA1c, lipid profiles, or systolic blood pressure. Diastolic blood pressure decreased slightly, while HOMA-IR showed a non-significant downward trend. **Conclusions**: Short-term oral NMN or NMN-related supplementation showed favorable tolerability, with no clear increase in adverse events or hepatic biochemical abnormalities. Broad metabolic benefits were not evident, but changes in diastolic blood pressure and HOMA-IR suggest preliminary vascular-metabolic signals, especially in older adults or people with early metabolic risk. Larger and longer trials should confirm efficacy and long-term safety. This review was registered in PROSPERO (CRD420261382497).

## 1. Introduction

Body weight, glucose metabolism, lipid profiles, blood pressure, and liver enzymes are commonly used clinical indicators for evaluating metabolic health in adults. Traditionally, these indicators are often interpreted within relatively separate disease frameworks, such as obesity, dyslipidemia, type 2 diabetes, fatty liver disease, or hypertension. However, metabolic abnormalities often emerge gradually before explicit diagnostic thresholds are reached. Longitudinal studies have shown that changes in glycemia, insulin sensitivity, and insulin secretion may occur years before the diagnosis of type 2 diabetes [1]. In addition, insulin resistance [2,3], chronic low-grade inflammation [4], metabolic dysfunction-associated fatty liver disease [5,6,7], disturbances in NAD+ homeostasis and mitochondrial function [8,9], and impaired inter-organ metabolic crosstalk [10] may all contribute to the continuous progression among obesity, fatty liver disease, metabolic syndrome, type 2 diabetes, and cardiometabolic abnormalities. Therefore, body weight, glucose, lipids, liver enzymes, and blood pressure may be understood as interrelated phenotypes within the metabolic disease continuum rather than as isolated endpoints [11].

NMN is an important precursor of NAD+. NAD+ is a key coenzyme in redox reactions and a substrate for multiple NAD+-dependent enzymes. It participates in mitochondrial energy metabolism, DNA repair, oxidative stress responses, inflammatory regulation, and metabolic homeostasis [8,9,12]. Animal studies have shown that NMN supplementation can increase NAD+ availability and may exert favorable effects in models of abnormal glucose metabolism, age-related functional decline, and vascular dysfunction [13,14,15]. These basic studies provide biological rationale for human research on NMN, but animal and mechanistic findings cannot be directly equated with evidence of clinical efficacy. Recent reviews have consistently concluded that although oral NMN supplementation effectively increases NAD^+^ availability and appears to be well tolerated in humans, current clinical evidence remains limited by small sample sizes, heterogeneous study populations, and short intervention durations. Therefore, further evidence synthesis based on randomized controlled trials is needed to better define its clinical efficacy and safety [16,17].

Despite the biological plausibility of NMN, results from adult randomized controlled trials have been inconsistent, and previous reviews have noted that human clinical evidence remains in an accumulating stage [18]. Recent reviews have similarly emphasized that oral NAD^+^ precursor supplementation can increase NAD^+^ availability in humans, but current clinical evidence remains constrained by small sample sizes, short follow-up periods, heterogeneous populations, and inconsistent efficacy endpoints [17,19,20]. More broadly, a human-focused review further noted that evidence for age-related NAD^+^ decline in humans remains limited across tissues, that the clinical efficacy of NAD^+^ precursor supplementation has not yet been consistently demonstrated, and that rodent-based mechanistic findings should not be directly extrapolated to humans [21]. Some studies have observed that NMN or MIB-626, a microcrystalline β-nicotinamide mononucleotide formulation, increases NAD+-related metabolites [22,23,24,25], or showed signals of improvement in insulin sensitivity [26], aerobic capacity [27], arterial stiffness trends [23], and blood pressure or vascular function [28] in selected populations. However, in healthy adults, older adults, patients with type 2 diabetes, or overweight/obese populations, the effects of NMN on body weight, glucose and lipid metabolism, liver enzymes, physical function, and liver fat have not reached consistent conclusions [25,29,30,31,32,33,34,35]. These differences may be related to study population, baseline metabolic status, age, sex, BMI, dose, formulation type, intervention duration, endpoint selection, and statistical power. In particular, BMI and body weight do not fully capture metabolic heterogeneity, because individuals with similar degrees of obesity may differ substantially in visceral adiposity, ectopic fat deposition, insulin sensitivity, and cardiometabolic risk [36].

At the same time, regulatory safety assessment of β-NMN has received increasing attention. β-NMN refers to the β-anomer of nicotinamide mononucleotide and is the form generally referred to as NMN in dietary supplement and human trial contexts; therefore, NMN is used in this review to refer to β-NMN unless otherwise specified. The European Food Safety Authority recently evaluated the safety of chemically synthesized β-NMN as a novel food and as a source of niacin in dietary supplements. It concluded that, under the proposed conditions of use, β-NMN is safe for adult dietary supplementation up to 300 mg/day, excluding pregnant and lactating women [37]. This regulatory opinion provides an important background for the safety of NMN as a dietary supplement, but it does not demonstrate metabolic efficacy or disease-modifying effects.

**Figure 1:**
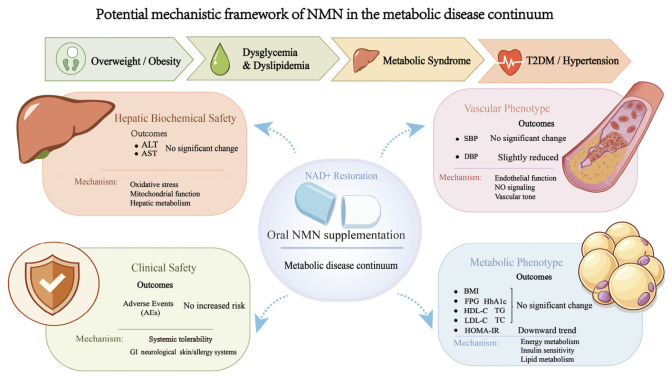
Hypothetical interpretive framework linking oral NMN or NMN-related supplementation, NAD+ biology, and metabolism-related clinical outcomes. This figure is intended to provide a conceptual framework only and does not imply that the mechanisms shown were directly confirmed by the present meta-analysis. Clinical outcomes shown in the figure summarize the pooled findings, whereas mechanistic pathways remain hypothetical and require further validation. Abbreviations: SBP, systolic blood pressure; DBP, diastolic blood pressure; ALT, alanine aminotransferase; AST, aspartate aminotransferase; BMI, body mass index; FPG, fasting plasma glucose; HDL-C, high-density lipoprotein cholesterol; LDL-C, low-density lipoprotein cholesterol; TC, total cholesterol; TG, triglyceride; AEs, adverse events.

Previous reviews have mainly summarized the biological rationale or anti-aging potential of NMN, whereas comprehensive evaluations integrating safety outcomes with metabolism-related clinical indicators from randomized controlled trials remain limited. Therefore, the present review primarily focuses on the safety profile of oral NMN supplementation while simultaneously evaluating its metabolism-related clinical effects [17,38]. Therefore, the primary aim of this systematic review and meta-analysis was to evaluate the short-term safety and tolerability of oral NMN as a dietary supplement based on evidence from adult randomized controlled trials, focusing on total adverse events, serious adverse events, withdrawals due to adverse events, system-specific adverse events, and hepatic biochemical indicators. Recent NMN-focused meta-analyses have primarily evaluated glucose and lipid outcomes, whereas safety events, hepatic biochemical indicators, and vascular phenotypes have been less comprehensively integrated within a single adult RCT-based synthesis [39,40]. On the basis of safety evaluation, this study further examined the effects of NMN on metabolism-related clinical indicators, including body weight, BMI, glucose metabolism, lipid profiles, and blood pressure. The metabolic disease continuum is used in this article only as an interpretive framework to understand the potential relationships among different metabolic phenotypes; it is not used as an eligibility criterion and should not be used to infer that NMN has therapeutic effects for any specific metabolic disease. The conceptual framework used to organize the clinical outcomes and hypothetical mechanistic links is presented in Figure 1.

## 2. Materials and Methods

### 2.1. Protocol and Reporting Guideline

**Figure 2:**
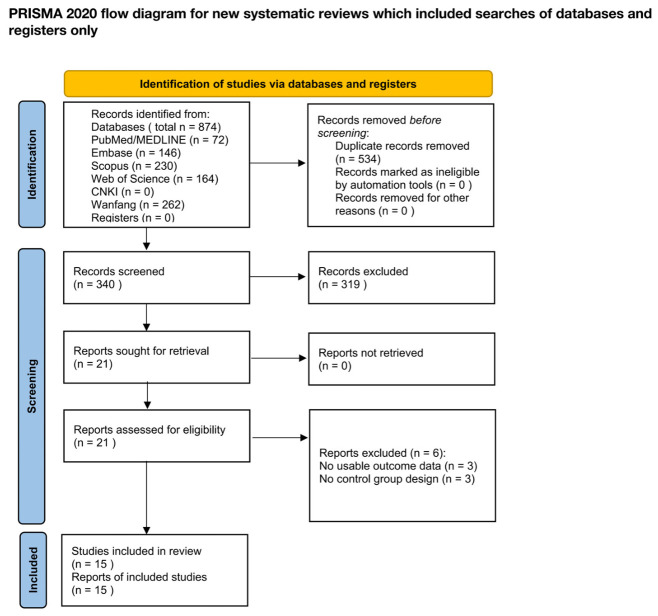
PRISMA 2020 flow diagram for study selection. Note: The total database records identified (n = 874) represent the sum of database-specific records before duplicate removal.

This systematic review and meta-analysis was conducted and reported in accordance with the PRISMA 2020 statement [41]. The review protocol was registered in PROSPERO before data extraction (registration number: CRD420261382497), and the protocol is accessible through the PROSPERO database using this registration number. After registration, the protocol was amended to include safety and tolerability outcomes and to define safety as the primary interpretive focus of the review, whereas glycemic, blood pressure, and other metabolism-related outcomes were retained as secondary or exploratory outcomes. This amendment was made because NMN is mainly used as a dietary supplement, for which short-term safety and tolerability are clinically and regulatorily important. The amendment did not change the target population, intervention, comparator framework, or randomized controlled trial design eligibility. The completed PRISMA 2020 checklist is provided in Supplementary Table S4, and the study selection process is presented in the PRISMA 2020 flow diagram in Figure 2.

### 2.2. Search Strategy and Eligibility Criteria

PubMed, Embase, Scopus, Web of Science, CNKI, and Wanfang databases were systematically searched from inception to 13 May 2026, without language restrictions. Search terms included NMN-related expressions such as “nicotinamide mononucleotide”, “NMN”, and the corresponding Chinese-language terms used for searches in CNKI and Wanfang, combined with randomized trial-related terms such as “randomized controlled trial”, “randomised controlled trial”, “placebo”, “clinical trial”, and the corresponding Chinese-language terms for randomized controlled trials. The complete database-specific search strategies, including the original Chinese search terms and their English meanings, are provided in Supplementary Table S1. Eligibility criteria were defined according to the PICOS framework. Participants were adults aged 18 years or older, without restrictions on sex, health status, baseline BMI, metabolic risk, or comorbidities. The intervention was oral NMN or an NMN-related preparation, with NMN as the single active intervention; no restrictions were imposed on dose, formulation, or intervention duration. Comparators included placebo, blank control, usual lifestyle control, or a control without NMN but with the same background intervention. Studies were required to report at least one hepatic biochemical, metabolic, blood pressure, or safety outcome, including ALT, AST, body weight, BMI, fasting plasma glucose, glycated hemoglobin, HDL-C, LDL-C, total cholesterol, triglycerides, systolic blood pressure, diastolic blood pressure, total adverse events, serious adverse events, withdrawals due to adverse events, or system-specific adverse events. Eligible study designs were parallel randomized controlled trials, including blinded or open-label randomized trials, provided that the comparator allowed the independent effect of NMN to be evaluated.

Exclusion criteria included animal or cell studies, observational studies, single-arm studies, nonrandomized intervention studies, case reports, reviews, conference abstracts without extractable data, studies in non-adult populations, studies of non-oral NMN, crossover trials without extractable parallel-group data, combined-intervention studies in which the independent effect of NMN could not be separated, duplicate reports, and studies in which outcome data could not be extracted or converted. If multiple reports described the same trial, the report with the most complete sample size, follow-up duration, or outcome data was selected, and duplicate counting was avoided.

The metabolic disease continuum framework was not used to restrict the study population. This framework was used only to explain potential relationships among different metabolic phenotypes and why NMN effects may differ across outcomes. Therefore, this review focuses on whether short-term oral NMN supplementation affects adult safety and metabolism-related clinical indicators, rather than evaluating whether NMN treats any specific metabolic disease.

### 2.3. Outcomes and Mechanism-Phenotype Mapping

Outcomes were divided into four domains:

1. Safety outcomes: total adverse events, serious adverse events, withdrawals due to adverse events, and system-specific adverse events.
2. Hepatic biochemical indicators: ALT and AST.
3. Metabolic phenotypes: body weight, BMI, fasting plasma glucose, glycated hemoglobin, HDL-C, LDL-C, total cholesterol, triglycerides, and HOMA-IR.
4. Vascular phenotypes: systolic and diastolic blood pressure.

Mechanistic indicators, including NAD+ metabolites, insulin sensitivity, inflammatory markers, oxidative stress markers, endothelial function, arterial stiffness, gut microbiota, and mitochondrial function, were descriptively extracted when available. The mechanism–phenotype interpretive framework corresponding to these outcome domains is summarized in Table 1.

### 2.4. Data Extraction, Handling of Multi-Arm Studies, and Statistical Analysis

Two investigators independently screened the literature and extracted data, with disagreements resolved through discussion. Extracted information included study characteristics, participant characteristics, intervention details, comparator information, outcome data, adverse events, and mechanistic indicators. If a study reported multiple post-baseline time points, data at the end of the intervention were preferentially extracted.

Continuous outcomes were converted to uniform units when necessary: ALT and AST in U/L, body weight in kg, BMI in kg/m2, glucose and lipids in mg/dL, and blood pressure in mmHg. If standard deviations were missing, they were estimated according to methods recommended in the Cochrane Handbook [45].

For multi-arm studies including multiple eligible NMN or MIB-626 dose groups sharing the same control group, continuous outcomes were handled by splitting the sample size of the shared control group evenly while retaining the same control mean and standard deviation [45]. This method preserves dose-specific information while avoiding double-counting of the shared control group. For binary safety outcomes, multiple NMN groups were combined and compared with the full control group.

Meta-analyses were performed using R software (version 4.5.2; R Foundation for Statistical Computing, Vienna, Austria), mainly with the meta package (version 8.3.0) and metafor package (version 5.0.1). Continuous outcomes were pooled as mean differences and 95% confidence intervals. All analyses used random-effects models. Heterogeneity was assessed using I^2^, tau2, and Cochran Q tests. I^2^ values of approximately 25%, 50%, and 75% were interpreted as low, moderate, and high heterogeneity, respectively. Binary safety outcomes were primarily pooled as risk differences with 95% confidence intervals. Because serious adverse events were rare, the Peto odds ratio was used as a supplementary analysis. For HOMA-IR, mean differences in change from baseline were preferentially used for exploratory pooled analysis. Considering that baseline levels and variability of HOMA-IR may differ across studies, a sensitivity analysis using standardized mean differences was further performed to assess direction and robustness. For multi-arm dose studies, the shared control group was handled by splitting the sample size to avoid double-counting the control group. Because the HOMA-IR analysis included a limited number of studies, the related results were interpreted as hypothesis-generating evidence.

Leave-one-out sensitivity analyses were performed for key outcomes and outcomes affected by multi-arm comparisons. When data allowed, subgroup analyses were conducted according to age, baseline metabolic risk status, and intervention duration. Because the number of independent studies was limited and some comparisons came from multi-arm studies with shared controls, formal multivariable meta-regression was not performed. For outcomes with at least 10 comparisons, funnel plots, Egger tests, and trim-and-fill methods were used to assess publication bias or small-study effects [46,47]. However, because the number of studies was close to the recommended minimum threshold and some comparisons were not fully independent, these results should be interpreted cautiously.

In addition, because one included study enrolled hospitalized adults with COVID-19 and acute kidney injury, acute illness, organ injury, hospitalization, and concomitant medications could have affected adverse event reporting. Therefore, a specific sensitivity analysis excluding this study was performed in the safety dataset.

### 2.5. Risk of Bias and Certainty of Evidence Assessment

Risk of bias was independently assessed by two independent reviewers using the Cochrane RoB 2 tool [48], with disagreements resolved through discussion.

The certainty of evidence for ALT, AST, body weight, BMI, fasting plasma glucose, glycated hemoglobin, HOMA-IR, LDL-C, triglycerides, systolic blood pressure, diastolic blood pressure, total adverse events, and serious adverse events was evaluated using the GRADE framework [49].

The certainty of evidence was downgraded when there were concerns regarding risk of bias, inconsistency, indirectness, imprecision, or publication bias. Indirectness was considered when the study population, intervention, or outcome did not directly correspond to a specific metabolic disease treatment question. Because some trials included healthy adults, physically active participants, or clinically distinct populations rather than patients with explicit metabolic disease, indirectness should be particularly considered when interpreting metabolic efficacy.

## 3. Results

### 3.1. Study Selection and Characteristics of Included Studies

The database search identified 874 records. After removal of 534 duplicates, 340 records were screened by title and abstract, and 319 were excluded. Twenty-one full-text reports were assessed, of which six were excluded: three because they did not provide usable data for the prespecified quantitative synthesis, and three because they lacked an eligible randomized concurrent control group. The excluded reports are listed in Supplementary Table S5. Finally, 15 randomized controlled trials were included. The study selection process is shown in Figure 2.

The included populations comprised safety or efficacy studies in healthy adults [22,29,35], middle-aged or older healthy adults [23,30,33], and healthy older adults [31,34]. Other populations included recreational runners [27], overweight or obese adults [24,25], individuals with prediabetes or type 2 diabetes [26,32], adults with mild hypertension [28], and one acute disease population, namely hospitalized adults with COVID-19 and acute kidney injury [50]. NMN doses ranged from 250 mg/day to 2000 mg/day. Intervention duration ranged from 14 days to 24 weeks. Some studies used MIB-626, a microcrystalline NMN-related preparation [24,25,50], whereas the remaining studies used ordinary free NMN. The basic characteristics of the included randomized controlled trials are summarized in Table 2.

The safety analysis included 10 trials with 383 participants, including 230 in the NMN group and 153 in the control group.

### 3.2. Risk of Bias Assessment

**Figure 3:**
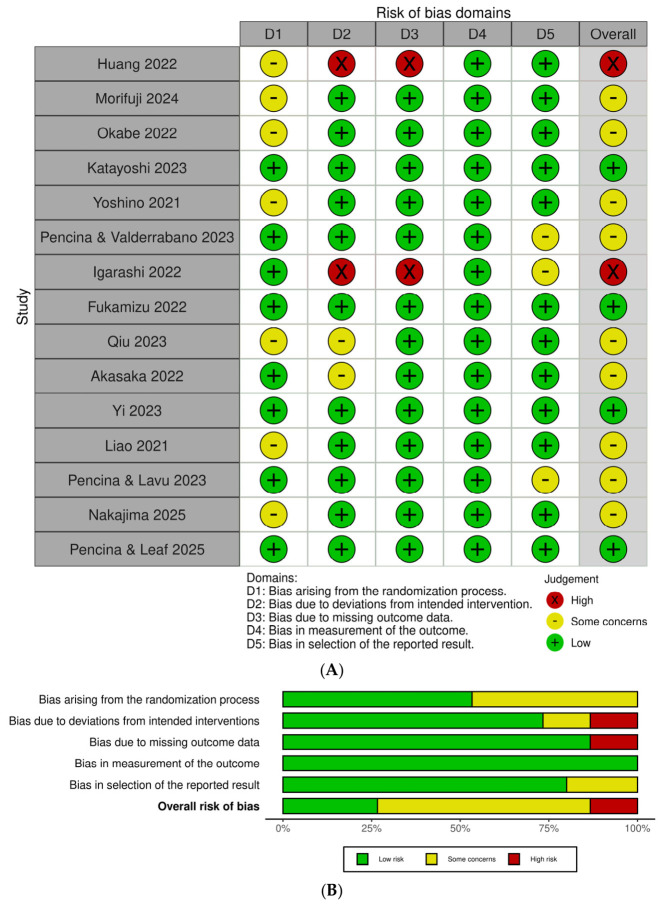
Risk of bias assessment of the included studies. (**A**). Risk of bias summary. (**B**). Risk of bias graph. Risk of bias assessment of the included studies using the Cochrane RoB 2 tool. (**A**) Risk of bias summary showing domain-level judgments for each included study. (**B**) Risk of bias graph showing the overall distribution of judgments across studies. Green indicates low risk of bias, yellow indicates some concerns, and red indicates high risk of bias. D1, bias arising from the randomization process; D2, bias due to deviations from intended interventions; D3, bias due to missing outcome data; D4, bias in measurement of the outcome; D5, bias in selection of the reported result. Trials judged as high risk of bias were retained in the main analysis, and their potential influence was considered in GRADE assessments and sensitivity analyses. References of included studies: [22,23,24,25,26,27,28,29,30,31,32,33,34,35,50].

Risk of bias was assessed using the Cochrane RoB 2 tool. Judgments for each study and each assessment domain are shown in Figure 3. Overall, most included trials were judged to have low risk of bias or some concerns. The main concerns arose from insufficient reporting of the randomization process, possible deviations from intended interventions in open-label or lifestyle-control studies, missing outcome data in small-sample studies, and incomplete reporting of prespecified outcomes in some trials. No single risk-of-bias domain was found to systematically affect the direction of the pooled results.

Considering that most included trials had small sample sizes and that several outcomes were not primary endpoints of the original studies, risk-of-bias assessments were incorporated into the GRADE interpretation of evidence certainty. Overall, risk of bias did not materially change the main conclusions, but it indicates that the exploratory diastolic blood pressure signal and rare-event safety outcomes should be interpreted cautiously.

### 3.3. Safety Outcomes

**Figure 4:**
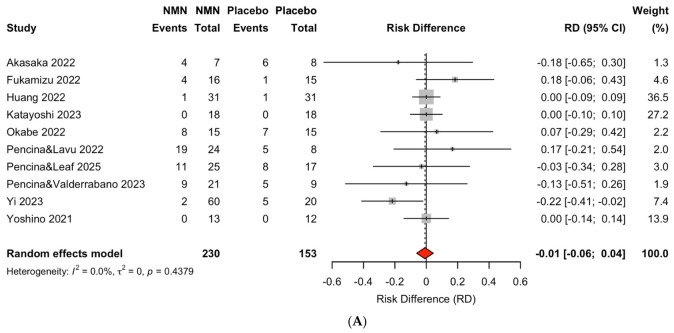

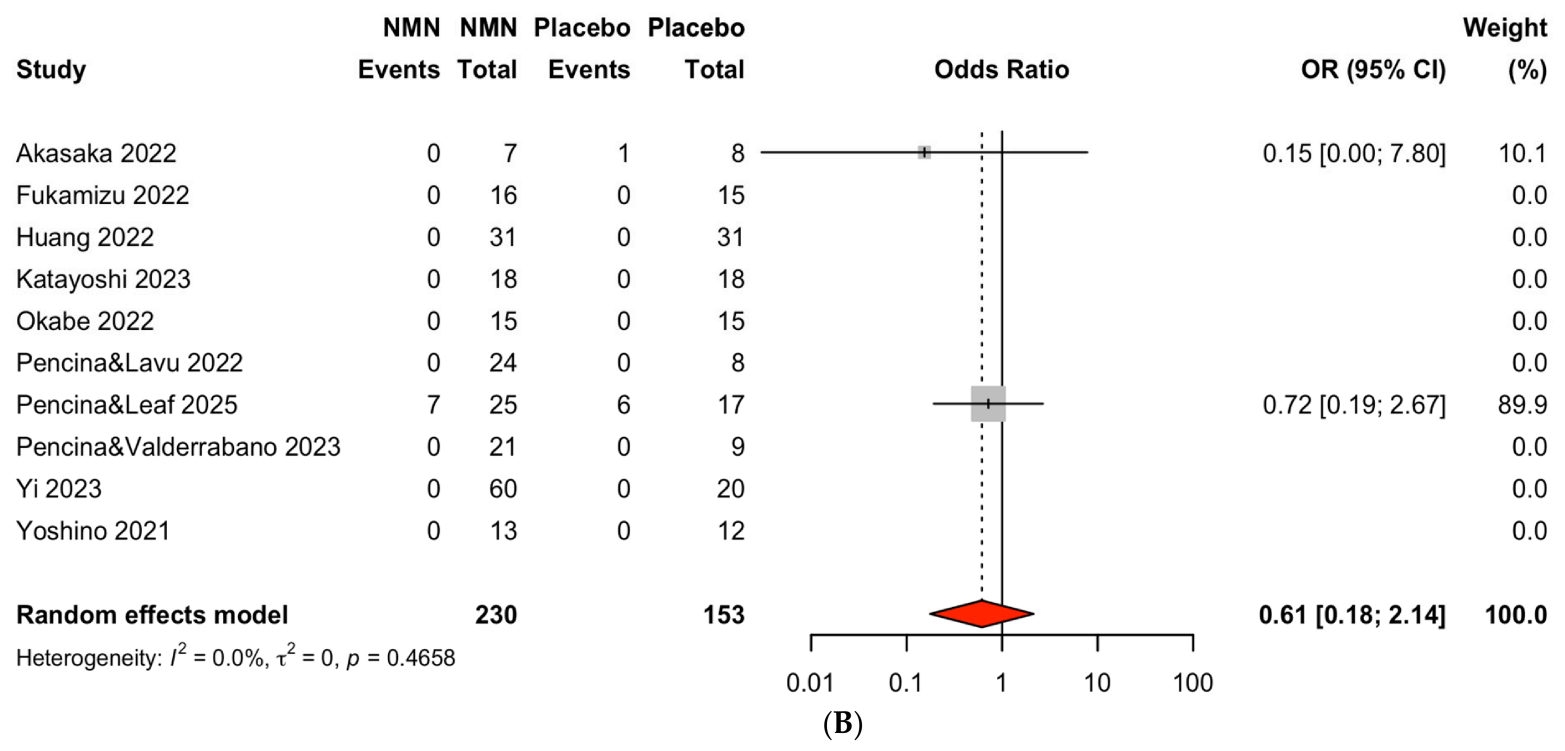
Forest plots for safety outcomes. (**A**) Any adverse events. (**B**) Serious adverse events. Pooled estimates were calculated using random-effects models. Supplementary Figures S1–S5 present system-specific adverse events and withdrawals due to adverse events. References of included studies: [22,23,24,25,26,29,30,32,33,50].

Because NMN is generally used as a dietary supplement rather than an established drug therapy, safety was treated as the primary interpretive focus of this study. The safety analysis included 10 randomized controlled trials with 383 participants. The remaining five studies were not included in the pooled safety analysis, mainly because they did not report extractable adverse event counts, provided only descriptive safety reports, or reported data in a format that could not be converted into binary pooled data. The main pooled safety findings are presented in Figure 4 and Table 3, with additional system-specific adverse event analyses shown in Supplementary Figures S1–S5.

There was no significant difference in total adverse events between the NMN and control groups:

RD = −0.008, 95% CI −0.061 to 0.045, *p* = 0.761, I^2^ = 0%.

Serious adverse events were rare. The primary risk difference analysis showed no significant difference between groups:

RD = −0.003, 95% CI −0.036 to 0.031, *p* = 0.878, I^2^ = 0%.

The supplementary Peto odds ratio analysis produced a consistent null-effect result:

Peto OR = 0.61, 95% CI 0.18 to 2.14, *p* = 0.443, I^2^ = 0%.

Withdrawals due to adverse events also did not differ significantly between groups:

RD = −0.001, 95% CI −0.036 to 0.034, *p* = 0.950, I^2^ = 0%.

System-specific adverse events, including gastrointestinal, nervous system, skin/allergic, and other events, did not show a significantly increased risk in the NMN group. In addition, ALT and AST were not significantly elevated, suggesting that no clear hepatic biochemical safety concern was observed in existing short-term trials.

Overall, current randomized controlled trials did not show evidence that short-term oral NMN supplementation increases the risk of adverse events or hepatic biochemical abnormalities, suggesting generally good tolerability. It should be emphasized that the current safety dataset has a limited sample size, short intervention duration, and few serious adverse events. Therefore, these results should not be interpreted as excluding potential rare, delayed, long-term, high-dose, or population-specific risks; such safety issues require further confirmation in future studies.

### 3.4. Hepatic Biochemical Indicators

The ALT analysis included 10 comparisons with 330 participants. NMN supplementation did not significantly alter ALT levels:

MD = −1.03 U/L, 95% CI −2.51 to 0.44, *p* = 0.169, I^2^ = 0%.

The AST analysis also included 10 comparisons with 330 participants. NMN supplementation did not significantly alter AST levels:

MD = −0.24 U/L, 95% CI −1.43 to 0.94, *p* = 0.686, I^2^ = 0%.

**Figure 5:**
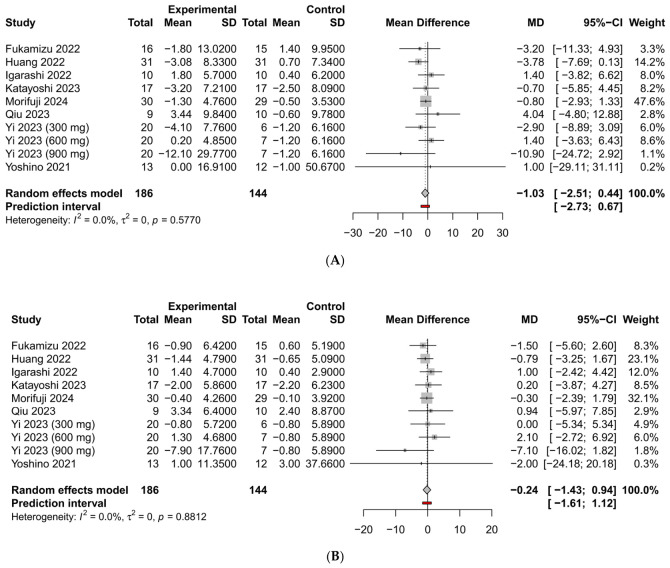
Effects of oral NMN or NMN-related supplementation on liver enzymes. (**A**) ALT. (**B**) AST. References of included studies: [23,26,28,29,30,31,33,34].

The forest plots for ALT and AST are shown in Figure 5A,B. These results do not support a clear liver enzyme-lowering or hepatoprotective effect of NMN. At the same time, no liver enzyme elevation signal was observed in short-term trials. Therefore, ALT and AST are more appropriately interpreted as evidence of short-term hepatic biochemical safety rather than as evidence of hepatic therapeutic benefit from NMN.

### 3.5. Metabolic Phenotypes

**Figure 6:**
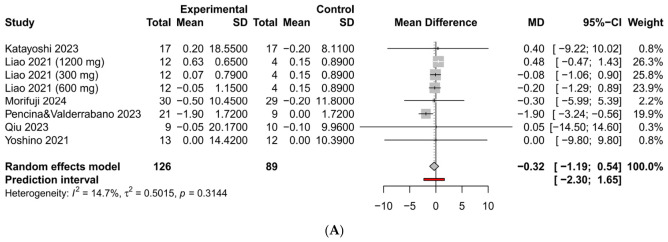

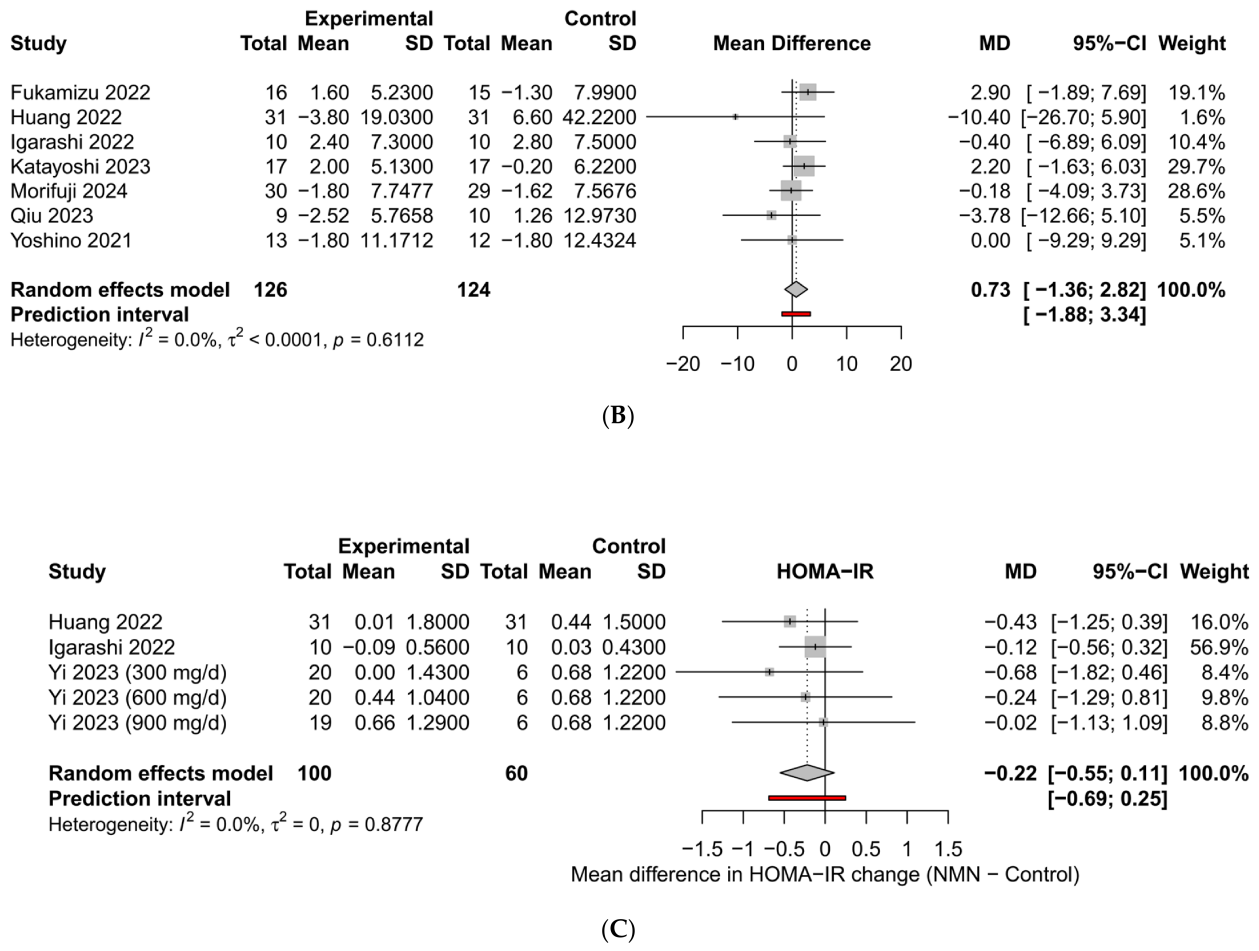
Effects of oral NMN or NMN-related supplementation on selected metabolic outcomes. (**A**) Body weight; (**B**) fasting plasma glucose; (**C**) HOMA-IR. Pooled estimates were calculated using random-effects models. The HOMA-IR analysis was exploratory and based on a limited number of comparisons; therefore, it should be interpreted as hypothesis-generating. Additional metabolic outcomes are presented in Supplementary Figures S6–S11, including BMI, HbA1c, HDL-C, LDL-C, total cholesterol, and triglycerides. References of included studies: [23,25,26,27,28,29,30,31,33,34].

On the basis of safety evaluation, this study further analyzed the effects of NMN on metabolism-related indicators within the metabolic disease continuum framework. The selected metabolic forest plots are shown in Figure 6A–C, and additional metabolic outcomes are presented in Supplementary Figures S6–S11.

NMN supplementation had no significant effect on body weight:

MD = −0.32 kg, 95% CI −1.19 to 0.54, *p* = 0.462, I^2^ = 14.7%.

BMI also did not change significantly:

MD = 0.02 kg/m2, 95% CI −0.14 to 0.18, *p* = 0.795, I^2^ = 0%.

Fasting plasma glucose did not change significantly:

MD = 0.73 mg/dL, 95% CI −1.36 to 2.82, *p* = 0.492, I^2^ = 0%.

Glycated hemoglobin was not significantly affected:

MD = 0.03%, 95% CI −0.04 to 0.10, *p* = 0.342, I^2^ = 22.4%.

HOMA-IR showed a mild improvement trend:

MD = −0.22, 95% CI −0.55 to 0.11, *p* = 0.19, I^2^ = 0.0%.

The HOMA-IR analysis included five comparisons, comprising Huang 2022 [30], Igarashi 2022 [31], and the 300, 600, and 900 mg/day dose subgroups from Yi 2023 [33], with a total of 160 participants. The random-effects model showed a downward trend in HOMA-IR in the NMN group compared with the control group, with a pooled effect of MD = −0.22 (95% CI −0.55 to 0.11), I^2^ = 0.0%, suggesting that NMN may have a potentially favorable effect on insulin resistance-related functional metabolic phenotypes.

Dose subgroup analysis showed no significant subgroup difference (χ^2^ = 0.14, df = 2, *p* = 0.9344); intervention-duration subgroup analysis also showed no significant difference (χ^2^ = 0.46, df = 1, *p* = 0.4958). After using SMD for sensitivity analysis, the direction of the result remained consistent (SMD = −0.24, 95% CI −0.58 to 0.09, I^2^ = 0.0%). Overall, the HOMA-IR results may be viewed as a directionally consistent, hypothesis-generating signal related to insulin sensitivity.

Regarding lipid indicators, NMN supplementation had no significant effect on HDL-C, LDL-C, total cholesterol, or triglycerides. LDL-C showed moderate heterogeneity, mainly influenced by one study, but the overall negative conclusion remained unchanged after sensitivity analysis.

Taken together, current randomized controlled trial evidence indicates that short-term NMN supplementation does not produce broad effects on body weight, BMI, conventional glucose metabolism indicators, or traditional lipid profiles. However, a consistent downward trend was observed in the insulin resistance index (HOMA-IR), suggesting a possible effect of NMN on insulin sensitivity. This finding warrants further validation in larger and longer studies. Based on current evidence, NMN should not be regarded as an established treatment for metabolic diseases such as obesity, diabetes, or dyslipidemia, but its preliminary signal in improving insulin resistance provides a promising direction for future research.

### 3.6. Blood Pressure Outcomes

The systolic blood pressure analysis included 11 comparisons with 339 participants. The overall effect was not statistically significant (MD = −0.89 mmHg, 95% CI −3.83 to 2.05, *p* = 0.553), with moderate between-study heterogeneity (I^2^ = 46.3%). After excluding the Fukamizu 2022 study [29], heterogeneity decreased to 0%, but the pooled effect remained statistically nonsignificant (MD = −2.00 mmHg, 95% CI −4.24 to 0.24, *p* = 0.081).

**Figure 7:**
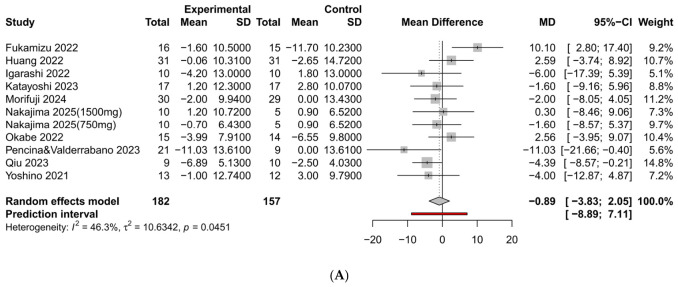

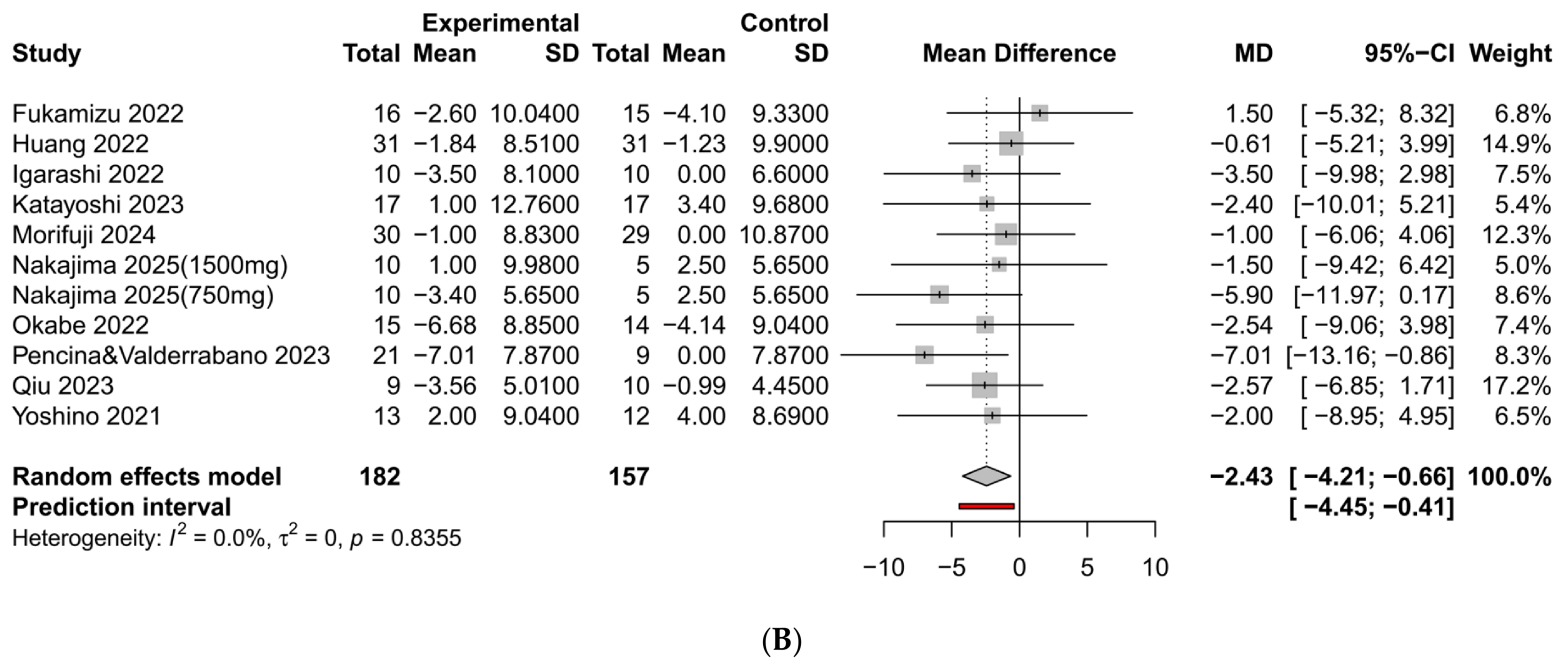
Effects of oral NMN or NMN-related supplementation on blood pressure. (**A**) Systolic blood pressure. (**B**) Diastolic blood pressure. Pooled estimates were calculated using random-effects models. Although diastolic blood pressure showed a small statistically significant reduction, this finding should be interpreted as exploratory because blood pressure was not the primary endpoint in most included trials and multiple outcomes were analyzed. References of included studies: [22,23,25,26,28,29,30,31,34,35].

The diastolic blood pressure analysis also included 11 comparisons with 339 participants. NMN supplementation was associated with a small but significant reduction in diastolic blood pressure (MD = −2.43 mmHg, 95% CI −4.21 to −0.66, *p* = 0.007, I^2^ = 0%). The forest plots for systolic and diastolic blood pressure are shown in Figure 7A,B. Leave-one-out sensitivity analyses supported the robustness of this finding: after sequentially excluding each study or independent comparison, pooled MDs ranged from −2.75 to −2.02 mmHg, all 95% confidence intervals remained below 0, and heterogeneity was consistently 0%. Even in the most conservative exclusion analysis (excluding Pencina & Valderrabano 2023 [25]), the effect remained statistically significant (MD = −2.02 mmHg, 95% CI −3.87 to −0.16, *p* = 0.033). These results suggest that the diastolic blood pressure signal was not driven by a single study or a single dose subgroup.

Notably, exploratory subgroup analyses, with the corresponding forest plots provided in Supplementary Figure S15A–F and numerical results summarized in Supplementary Table S6, identified a more pronounced and directionally consistent blood pressure-lowering effect of NMN in older and metabolically susceptible populations. For DBP, significant reductions were observed among participants aged 60 years or older (MD = −3.18 mmHg, 95% CI −6.19 to −0.16), individuals with metabolic risk, including overweight/obesity, prediabetes, or hypertension (MD = −3.61 mmHg, 95% CI −6.74 to −0.47), and trials with an intervention duration shorter than 10 weeks (MD = −2.63 mmHg, 95% CI −4.90 to −0.35). These effects were less evident in younger participants, healthy populations, and trials lasting 10 weeks or longer. For SBP, significant reductions were also observed among participants aged 60 years or older (MD = −4.40 mmHg, 95% CI −8.61 to −0.20) and in metabolic-risk populations (MD = −5.07 mmHg, 95% CI −8.64 to −1.51), whereas no comparable benefit was detected in healthy participants. Most formal tests for subgroup differences were not statistically significant; however, a significant subgroup difference was observed for SBP according to metabolic status. Overall, the consistency of these subgroup estimates strengthens the possibility that NMN-related vascular benefits may be particularly evident in older or metabolically vulnerable individuals, although these findings should be interpreted as exploratory.

It should be noted that most included trials did not designate blood pressure as a primary endpoint, and this study involved multiple outcome comparisons. Therefore, the diastolic blood pressure result should be interpreted as an exploratory vascular phenotype signal, suggesting that NMN may have potential vascular health benefits in older or metabolically at-risk populations; however, it cannot yet be considered evidence of an established antihypertensive effect of NMN. Future studies designed for high-risk populations and using blood pressure as a primary endpoint are warranted.

### 3.7. Descriptive Mechanistic Findings

The reporting of direct mechanistic indicators varied across trials. Some studies measured NAD+ metabolites, insulin sensitivity, muscle or physical function, arterial stiffness, and related biomarkers, but there was substantial heterogeneity in biological sample type, measurement method, time point, and reporting format. Therefore, these findings were mainly summarized descriptively. A recent randomized, open-label, placebo-controlled human study that included an NMN intervention arm showed that 14 days of NMN supplementation increased circulating NAD^+^ concentrations in healthy adults, with effects comparable to those observed for nicotinamide riboside. Ex vivo experiments further suggested that gut microbial conversion to nicotinic acid may contribute to the NAD^+^-boosting effects of NMN and nicotinamide riboside. Because this study primarily focused on NAD^+^ metabolomics and microbial metabolism rather than prespecified clinical metabolic or vascular endpoints, it should be interpreted only as supportive mechanistic evidence and not as direct evidence of NMN clinical efficacy [51].

The overall evidence pattern showed that NMN did not demonstrate broadly consistent improvements in conventional metabolic indicators such as body weight, FPG, HbA1c, and lipids. However, potentially beneficial mechanistic signals were observed in a small number of high-precision mechanistic trials. In particular, Yoshino 2021 [26] used the glucose clamp technique in overweight or obese postmenopausal women with prediabetes and observed improvements in peripheral insulin sensitivity-related indicators in the NMN group. This finding provides a mechanistic clue that NMN may affect skeletal muscle insulin action, although replication in other metabolic-risk populations is still needed because the study population was specific and the sample size was limited. This experimental result provides not only functional support for the downward trend observed in HOMA-IR but also direct functional clinical evidence for a potential mechanism by which NMN may improve insulin sensitivity.

It should be emphasized that, although only a limited number of glucose clamp experiments are available, such tests provide highly precise and direct functional indicators. Compared with conventional metabolic indicators, they can more reliably reflect improvements in peripheral tissue insulin action. Therefore, this finding may be regarded as a mechanistic signal of interest in current adult clinical research, whereas other conventional metabolic indicators still lack broad consistency.

### 3.8. Sensitivity Analyses

Leave-one-out sensitivity analyses were performed for key clinical and safety outcomes. The pooled estimates for ALT, AST, body weight, BMI, fasting plasma glucose, glycated hemoglobin, HDL-C, total cholesterol, triglycerides, diastolic blood pressure, and total adverse events remained generally stable after sequential exclusion of each study or independent comparison. LDL-C heterogeneity was mainly influenced by the Pencina & Valderrabano 2023 study [25], but the overall negative conclusion did not change after excluding this study. For systolic blood pressure, heterogeneity decreased after excluding Fukamizu 2022 [29], but the pooled effect remained statistically nonsignificant. The diastolic blood pressure reduction remained statistically significant in all leave-one-out analyses, supporting the internal consistency of this exploratory signal.

Detailed results of the leave-one-out sensitivity analyses are shown in Supplementary Table S3.

One included study enrolled hospitalized adults with COVID−19 and acute kidney injury [50]. Because acute illness, organ injury, hospitalization, and concomitant medications could influence adverse event reporting, a specific sensitivity analysis excluding this study was performed in the safety dataset. After excluding this study, all safety outcomes remained statistically nonsignificant, and heterogeneity did not change materially.

### 3.9. Publication Bias and Certainty of Evidence

Publication bias and small-study effects were assessed for outcomes with at least 10 comparisons. The funnel plot for ALT suggested mild asymmetry; however, the trim-and-fill analysis did not materially change the non-significant result. Funnel plots for AST, HDL-C, LDL-C, triglycerides, systolic blood pressure, diastolic blood pressure, and total adverse events did not show substantial asymmetry. Nevertheless, because the number of studies was close to the recommended minimum threshold and some comparisons were derived from multi-arm trials, these findings should be interpreted cautiously. Funnel plots and trim-and-fill analyses are presented in Supplementary Figures S12–S14.

The GRADE assessment showed moderate certainty of evidence for ALT, AST, body weight, BMI, fasting plasma glucose, triglycerides, and total adverse events; low certainty for glycated hemoglobin, LDL-C, systolic blood pressure, and diastolic blood pressure; and very low certainty for HOMA-IR and serious adverse events. Details are provided in Supplementary Table S2. Although diastolic blood pressure showed a statistically significant decrease, this result should be viewed as hypothesis-generating rather than confirmatory because of the limited sample size, indirectness, and the fact that blood pressure was not the primary endpoint in most trials. Similarly, although HOMA-IR showed a directionally consistent downward trend, the certainty of evidence was very low and should be interpreted as an exploratory signal related to insulin sensitivity.

## 4. Discussion

### 4.1. Main Findings

This systematic review and meta-analysis found no evidence from existing short-term randomized controlled trials in adults that oral NMN supplementation increases the risk of adverse events or hepatic biochemical abnormalities, suggesting generally good tolerability as a dietary supplement. ALT and AST did not increase significantly, and the risks of total adverse events, serious adverse events, withdrawals due to adverse events, and system-specific adverse events were not increased.

Regarding metabolism-related outcomes, NMN did not show consistent improvements in most conventional indicators. This pattern is broadly consistent with recent meta-analyses showing that short-term NMN supplementation does not produce robust improvements in conventional glucose or lipid outcomes despite increasing NAD^+^ availability [39,40]. Notably, HOMA-IR showed a directionally consistent downward trend, and diastolic blood pressure showed a small reduction, suggesting exploratory signals related to insulin sensitivity and vascular phenotypes. However, these results require validation in larger randomized trials in which the relevant phenotypes are primary endpoints.

This blood pressure signal is consistent with a recent NMN-focused meta-analysis of randomized controlled trials, which reported a modest reduction in resting diastolic blood pressure but no significant overall effect on systolic blood pressure. However, the present review extends previous work by integrating safety outcomes, hepatic biochemical indicators, broader metabolic phenotypes, and vascular outcomes within a single adult RCT-based synthesis [44].

### 4.2. Interpretation Within the Metabolic Disease Continuum Framework

The metabolic disease continuum framework helps explain the pattern of metabolic effects observed in this meta-analysis. This framework emphasizes that obesity-associated metabolic abnormalities can be understood as a dynamic continuum, and that metabolic dysfunction-associated fatty liver disease overlaps with metabolic syndrome, type 2 diabetes, and cardiometabolic risk [5,6,7]. Insulin resistance is an important basis of this continuum [2,3], and chronic low-grade inflammation [4] and ectopic fat deposition [2,3] can further drive the progression of metabolic abnormalities. Disturbances in NAD+ homeostasis may contribute to cellular metabolic imbalance by affecting processes such as energy metabolism and DNA repair [8,9], and impaired inter-organ metabolic crosstalk may also participate in the development and progression of metabolic diseases such as type 2 diabetes [10].

From this perspective, this study identified a noteworthy signal pattern: NMN supplementation did not show broad improvements across multiple conventional metabolic indicators, including body weight, BMI, fasting plasma glucose, glycated hemoglobin, lipid profiles, ALT, AST, and systolic blood pressure; however, diastolic blood pressure showed a small but statistically significant decrease (MD = −2.43 mmHg, 95% CI −4.21 to −0.66, *p* = 0.007, I^2^ = 0%), and this signal remained robust in leave-one-out sensitivity analyses. More importantly, HOMA-IR showed a directionally consistent downward trend (MD = −0.22, 95% CI −0.55 to 0.11; SMD = −0.24, 95% CI −0.58 to 0.09), with zero heterogeneity. Although this result did not reach statistical significance, its consistent direction and very low heterogeneity suggest that NMN may have a potentially favorable effect on insulin sensitivity, a functional metabolic phenotype. This interpretation is consistent with established metabolic physiology, because insulin resistance may precede overt abnormalities in fasting glucose or HbA1c and can occur before conventional metabolic biomarkers become clearly abnormal. Therefore, functional indicators of insulin sensitivity may be more sensitive than downstream clinical endpoints for detecting early metabolic effects of NAD^+^-boosting interventions [2,3].

Overall, current evidence does not support positioning NMN as a broad-spectrum metabolic intervention for unselected adult populations. However, the directionally consistent but exploratory signals observed for diastolic blood pressure and HOMA-IR suggest that NMN may have potential effects on vascular function and insulin resistance. This effect pattern is compatible with the metabolic disease continuum framework: insulin resistance and vascular dysfunction often appear before abnormalities in glycemic and lipid profiles and represent early components of metabolic disorder. Therefore, the absence of broad effects on relatively downstream clinical indicators such as fasting plasma glucose, HbA1c, and lipids does not exclude the possibility that NMN may act in early stages of the metabolic continuum or in selected subgroups.

Subgroup analyses in this study further support this interpretation, although these findings should be considered exploratory. In older adults aged 60 years or above, NMN supplementation was associated with reductions in both systolic blood pressure (MD = −4.40 mmHg, 95% CI −8.61 to −0.20) and diastolic blood pressure (MD = −3.18 mmHg, 95% CI −6.19 to −0.16). In individuals with metabolic risk, including overweight/obesity, prediabetes, or hypertension, reductions were also observed for systolic blood pressure (MD = −5.07 mmHg, 95% CI −8.64 to −1.51) and diastolic blood pressure (MD = −3.61 mmHg, 95% CI −6.74 to −0.47). Most formal tests for subgroup differences were not statistically significant, although a significant subgroup difference was observed for systolic blood pressure according to metabolic status. Taken together, these findings suggest that NMN-related vascular effects may be more apparent in selected populations, especially older or metabolically susceptible individuals, rather than universal across unselected healthy populations.

It should be acknowledged that the included trial populations were heterogeneous, spanning healthy adults, healthy older adults, overweight/obese individuals, people with prediabetes, patients with type 2 diabetes, adults with mild hypertension, and acutely hospitalized patients. Most original studies did not stratify participants by metabolic stage, insulin resistance status, or baseline NAD+ levels, limiting reliable identification of potential responder subgroups. Therefore, the HOMA-IR and blood pressure signals described above should be interpreted as directionally consistent but still unconfirmed exploratory evidence.

Taken together, within the current regulatory context of dietary supplementation or novel food use, the present findings should not be interpreted as supporting therapeutic or disease-treatment claims for established obesity, diabetes, fatty liver disease, dyslipidemia, or hypertension. However, its exploratory signals for HOMA-IR and diastolic blood pressure may help inform future research in selected populations with early metabolic risk, such as insulin resistance, prediabetes, or older age. Long-term, large-sample randomized controlled trials targeting these high-risk populations and using insulin sensitivity or vascular function as primary endpoints should be prioritized.

### 4.3. Interpretation of the Diastolic Blood Pressure Signal

The small reduction in diastolic blood pressure may suggest a potential vascular-related signal for NMN. This interpretation is biologically plausible because NAD^+^ metabolism is closely linked to endothelial function, vascular aging, oxidative stress, and blood pressure regulation. In particular, NAD^+^ depletion and altered activity of NAD^+^-consuming enzymes such as CD38 and PARPs may contribute to endothelial dysfunction and vascular injury, whereas NAD^+^ repletion has been proposed as a potential strategy to support vascular homeostasis [38,43,52]. This pattern is broadly consistent with recent meta-analyses showing that short-term NMN supplementation does not produce robust improvements in conventional glucose or lipid outcomes despite increasing NAD^+^ availability [39,40].

Diastolic blood pressure is influenced by peripheral vascular resistance, endothelial function, vascular tone, and autonomic regulation. Basic research has shown that SIRT1 promotes endothelium-dependent vasodilation by activating endothelial nitric oxide synthase [53]; NAD+ metabolism is related to cardiovascular aging and disease [52]. CD38 pathways associated with NAD+ decline may participate in age-related mitochondrial dysfunction [54], and NMN supplementation improves microvascular endothelial function and neurovascular coupling responses in aged animals [55]. In addition, hypertension-related research suggests that CD38 upregulation and NAD+ depletion may contribute to blood pressure elevation and vascular injury [28].

However, this interpretation remains hypothesis-generating. The included trials generally did not systematically measure endothelial function, nitric oxide bioavailability, central arterial pressure, pulse wave velocity, ambulatory blood pressure, or vascular inflammatory markers. Therefore, this meta-analysis cannot establish a clear mechanistic link between NMN supplementation and diastolic blood pressure reduction.

Notably, the reduction in diastolic blood pressure was not accompanied by consistent improvements in body weight, glycemic outcomes, or lipid outcomes. This pattern does not support interpreting the diastolic blood pressure reduction as a secondary consequence of broad metabolic improvement. Instead, it is better regarded as a potential vascular phenotype signal that needs further validation in trials specifically designed to assess blood pressure and vascular function.

Leave-one-out sensitivity analyses strengthened the internal consistency of the diastolic blood pressure finding. After sequentially excluding each study or independent comparison, the pooled effect remained statistically significant, with MDs ranging from −2.75 to −2.02 mmHg. However, the effect size was small, and in the most conservative leave-one-out analysis, the confidence interval was close to the null line. Therefore, the clinical significance of this diastolic blood pressure reduction remains to be explored.

### 4.4. Mechanistic Interpretation of Exploratory Signals

Beyond the vascular mechanisms discussed above, NAD^+^-related mitochondrial pathways may provide additional biological context for the exploratory signals observed in HOMA-IR and diastolic blood pressure. NMN is a nicotinamide-derived precursor of NAD^+^, which participates in cellular redox reactions, mitochondrial energy metabolism, and NAD^+^-dependent signaling pathways, including those involving sirtuins, PARPs, and CD38 [8,28]. Recent reviews have emphasized that NAD^+^ replenishment may influence mitochondrial oxidative metabolism, mitochondrial quality control, mitophagy, oxidative stress responses, and cellular resilience [17,42]. However, whether these mechanistic changes translate into clinically meaningful metabolic benefits in humans remains uncertain because adequately powered mechanistic trials are still limited.

These mechanisms may be relevant to the HOMA-IR signal observed in the present meta-analysis. In a randomized trial involving overweight or obese postmenopausal women with prediabetes, NMN supplementation improved skeletal muscle insulin sensitivity and insulin-signaling-related pathways [26]. This finding suggests that NAD^+^ precursor supplementation may preferentially affect functional metabolic phenotypes in selected populations, even when conventional downstream biomarkers such as body weight, fasting plasma glucose, HbA1c, or lipid profiles show no broad improvement. Nevertheless, the pooled HOMA-IR result in the present study was not statistically significant and should therefore be regarded as hypothesis-generating.

NAD^+^-related pathways may also be relevant to vascular regulation. NAD^+^ metabolism has been linked to endothelial function, vascular aging, arterial stiffness, inflammation, oxidative stress, and blood pressure regulation [43]. Evidence from hypertensive patients and experimental models suggests that CD38 upregulation may contribute to NAD^+^ depletion and vascular dysfunction, whereas NMN supplementation may increase NAD^+^ availability and improve blood pressure or vascular function [28]. These findings provide a possible biological explanation for the small reduction in diastolic blood pressure observed in this meta-analysis, but they do not establish causality.

These mechanistic interpretations should be considered cautiously. Most included trials did not directly assess mitochondrial respiration, oxidative phosphorylation capacity, mitochondrial biogenesis, mitophagy, tissue-specific NAD^+^ pools, endothelial nitric oxide bioavailability, or vascular mitochondrial function. Therefore, the present meta-analysis cannot determine whether mitochondrial or vascular NAD^+^-related mechanisms were responsible for the observed changes in HOMA-IR or diastolic blood pressure. Future randomized controlled trials should incorporate mitochondrial and NAD^+^-related biomarkers, together with insulin sensitivity and vascular function endpoints, to clarify whether NMN affects cardiometabolic phenotypes through mitochondrial energy metabolism or vascular-metabolic regulation.

### 4.5. Clinical Implications

From a clinical and regulatory perspective, NMN is currently better positioned as a candidate metabolic health-supporting dietary supplement with short-term tolerability evidence and biological plausibility, rather than as an established disease treatment. Current evidence therefore supports positioning NMN primarily as a dietary supplement with promising biological plausibility rather than as an evidence-based therapeutic intervention for established metabolic diseases. This distinction is important because improvements in circulating NAD^+^ concentrations do not necessarily translate into clinically meaningful benefits across multiple metabolic endpoints, particularly when evidence is derived from relatively small and short-term randomized trials [17,20]. Existing randomized controlled trial evidence has not shown broad and consistent therapeutic improvements in conventional metabolic indicators such as body weight, glucose, lipids, hepatic biochemical markers, or systolic blood pressure. Therefore, NMN should not currently be considered an alternative treatment for obesity, diabetes, fatty liver disease, dyslipidemia, or hypertension.

However, from the perspective of mechanisms and functional metabolic phenotypes, NMN, as an NAD+ precursor, may influence insulin signaling and vascular function by supporting NAD+ homeostasis, mitochondrial function, and energy metabolism. In this study, the downward trend in HOMA-IR and the small reduction in diastolic blood pressure suggest exploratory signals related to insulin sensitivity and vascular-metabolic regulation. Therefore, NMN may be further investigated as a candidate adjunctive intervention in populations with early metabolic abnormalities, insulin resistance, or age-related NAD+ decline, but its glucose-lowering or antihypertensive effects still require confirmation in larger and longer randomized controlled trials. Lifestyle intervention, weight management, and guideline-recommended glucose-lowering, lipid-lowering, and antihypertensive therapies remain the foundation of metabolic disease prevention and treatment. In individuals with prediabetes or high-risk profiles, lifestyle intervention has randomized trial evidence supporting its ability to reduce the incidence of type 2 diabetes [56,57].

### 4.6. Strengths and Limitations

This review has several strengths. First, it systematically synthesized evidence from parallel randomized controlled trials of oral NMN supplementation in adults and evaluated multiple clinically relevant outcomes, including safety, hepatic biochemical indicators, metabolic indicators, and blood pressure. Second, it interpreted these outcomes within the metabolic disease continuum framework, helping avoid treating body weight, glucose, lipids, liver enzymes, and blood pressure as entirely isolated endpoints. Third, multiple sensitivity analyses were used to assess robustness, and the potential influence of an acute disease population on safety outcomes was considered separately.

This study also has limitations. Consistent with previous reviews, the current evidence base remains dominated by small, short-term trials, which limits conclusions regarding long-term efficacy, rare adverse events, and population-specific safety signals [17,19,20]. The number of included studies and participants was small, and most trials had short intervention durations. Study populations ranged from healthy adults to metabolic-risk populations and one acute disease population, leading to some indirectness. NMN dose, formulation, and intervention duration were also inconsistent; some studies used MIB-626, whose pharmacokinetics and clinical effects may not be fully equivalent to those of ordinary free NMN [24,25,50]. In addition, existing safety data still cannot exclude rare, delayed, long-term, high-dose, or population-specific risks.

Another important limitation is that most original studies did not stratify participants according to their position within the metabolic disease continuum and rarely systematically assessed visceral fat, liver fat content, insulin sensitivity, inflammatory markers, mitochondrial function, endothelial function, or NAD+ metabolic status. Although current evidence is insufficient to systematically distinguish NMN effects across different metabolic stages or mechanistic phenotypes, the directionally consistent insulin sensitivity and vascular phenotype signals suggest potential benefit in selected high-risk individuals.

### 4.7. Future Research Directions

Future trials should first strengthen the safety evidence base for NMN as a dietary supplement. Larger randomized controlled trials with longer follow-up are needed to evaluate long-term tolerability, dose–safety relationships, hepatic and renal biochemical safety, drug interactions, and adverse events in older adults, patients with metabolic disease, and people with chronic comorbidities. Future studies should also incorporate standardized assessment of circulating NAD^+^ metabolites and mechanistic biomarkers to facilitate comparisons across trials and to better identify patient subgroups that may derive the greatest benefit from NAD^+^-boosting interventions [16,17,42].

On the basis of safety, future research should implement clearer population stratification, especially focusing on different stages of metabolic risk, including overweight or obesity, central obesity, insulin resistance, prediabetes, metabolic syndrome, fatty liver disease, type 2 diabetes, and hypertension.

Endpoint selection should match the disease stage. Future trials may include HOMA-IR, oral glucose tolerance testing, continuous glucose monitoring, hyperinsulinemic-euglycemic clamp, body composition, visceral fat, liver fat content, vascular function, and NAD+ metabolites as mechanistic or functional endpoints. They should also compare different doses, formulation types, and intervention durations to determine whether NMN can produce reproducible metabolic or vascular benefits in selected metabolic-risk populations.

## 5. Conclusions

This study shows that, in existing adult randomized controlled trials, short-term oral NMN supplementation did not show evidence of increased risks of adverse events, serious adverse events, withdrawals due to adverse events, or elevated hepatic biochemical indicators, suggesting generally good tolerability as a dietary supplement.

Current evidence does not show broad and consistent improvements in conventional metabolic indicators such as body weight, BMI, glycemia, lipids, or systolic blood pressure. HOMA-IR showed a directionally consistent downward trend, and diastolic blood pressure showed a small reduction with relatively stable sensitivity analyses, suggesting exploratory signals related to insulin sensitivity and vascular phenotypes. Considering differences across included studies in sample size, intervention duration, population characteristics, dose, and formulation, the current results should be understood as defining the boundaries of NMN clinical application and future research directions rather than negating its potential value.

Overall, NMN may be regarded as a candidate supportive intervention with clear biological plausibility, good short-term tolerability, and potential exploratory vascular-metabolic regulatory signals. Future larger, longer, and more mechanistically stratified randomized controlled trials should focus on evaluating its clinical value in populations with age-related NAD+ decline, early metabolic abnormalities, prediabetes, impaired vascular function, and other selected metabolic-risk states. In particular, future studies should include mitochondrial function and NAD^+^-related mechanistic endpoints to determine whether the exploratory signals observed for HOMA-IR and blood pressure are linked to changes in mitochondrial energy metabolism, endothelial function, or broader vascular-metabolic regulation.

## Figures and Tables

**Table 1:** Mechanism–phenotype interpretive framework for NMN supplementation in the metabolic disease continuum.

Mechanistic Domain	Relevant Tissues/ Organs	Pooled Clinical Outcomes	Interpretive Rationale	Representative Supporting Reference(s)
NAD+ metabolism, energy homeostasis, and insulin sensitivity	Skeletal muscle, liver, adipose tissue, pancreas	Body weight, BMI, FPG, HbA1c, HOMA-IR, glucose clamp-derived insulin sensitivity	To assess whether NMN supplementation may influence adiposity-related phenotypes, glucose homeostasis, and insulin sensitivity	[8,9,26,42]
Hepatic biochemical safety	Liver	ALT, AST	To evaluate short-term hepatic biochemical safety profiles	[22,29,35,37]
Lipid metabolism	Adipose tissue, liver, skeletal muscle	HDL-C, LDL-C, TC, TG	To assess changes in conventional lipid parameters	[8,39,40]
Vascular function and blood pressure phenotypes	Vascular system, kidney, autonomic nervous system	SBP, DBP	To examine potential vascular and hemodynamic effects	[23,28,43,44]
Clinical safety and tolerability	Gastrointestinal, neurological, dermatological, and other systems	Total AEs, SAEs, withdrawals due to AEs, system-specific AEs	To evaluate overall clinical safety and tolerability	[22,29,33,35,37]

Note for Table 1: This table outlines the conceptual framework linking proposed NMN-related mechanistic domains with the pooled clinical outcomes evaluated in this meta-analysis.

**Table 2:** Basic characteristics of the included randomized controlled trials.

Study (Year, Country)	Design	Population/Metabolic Profile	N (I/C)	Male, n (%)	Age (Years)	BMI (kg/m^2^)	Duration	Intervention/ Control	Reference(s)
Akasaka 2023 (Japan)	RCT, DB, PC	Older men with T2DM and impaired physical performance; T2DM/frailty	8 vs. 8	8/8 (100.0%) vs. 8/8 (100.0%)	83.0 ± 6.7 vs. 79.3 ± 6.0	NR	24 weeks	NMN 250 mg/day vs. placebo	[32]
Katayoshi 2023 (Japan)	RCT, DB, PC	Healthy middle-aged adults aged 40–59 years; healthy	18 vs. 18	8/18 (44.4%) vs. 6/18 (33.3%)	48.1 ± 5.4 vs. 47.9 ± 5.5	21.9 ± 4.3 vs. 21.7 ± 2.3	12 weeks	NMN 250 mg/day vs. placebo	[23]
Liao 2021 (China)	RCT, DB, PC	Healthy recreational runners; healthy/physically active	12 vs. 12	10/12 (83.3%) vs. 10/12 (83.3%)	37.0 ± 5.7 vs. 36.1 ± 6.0	22.3 ± 3.2 vs. 22.0 ± 2.6	6 weeks	NMN 300 mg/day + exercise vs. placebo + exercise	[27]
Liao 2021 (China)	RCT, DB, PC	Healthy recreational runners; healthy/physically active	12 vs. 12	10/12 (83.3%) vs. 10/12 (83.3%)	35.5 ± 6.1 vs. 36.1 ± 6.0	21.8 ± 2.9 vs. 22.0 ± 2.6	6 weeks	NMN 600 mg/day + exercise vs. placebo + exercise	[27]
Liao 2021 (China)	RCT, DB, PC	Healthy recreational runners; healthy/physically active	12 vs. 12	10/12 (83.3%) vs. 10/12 (83.3%)	33.5 ± 6.6 vs. 36.1 ± 6.0	21.9 ± 1.6 vs. 22.0 ± 2.6	6 weeks	NMN 1200 mg/day + exercise vs. placebo + exercise	[27]
Pencina & Valderrabano 2023 (USA)	RCT, DB, PC	Overweight or obese middle-aged and older adults; overweight/obesity	21 vs. 9	11/21 (52.4%) vs. 5/9 (55.6%)	60.9 ± 8.91 vs. 64.3 ± 7.63	29.1 ± 3.57 vs. 29.5 ± 3.79	28 days	MIB-626 2000 mg/day vs. placebo	[25]
Morifuji 2024 (Japan)	RCT, DB, PC	Healthy older adults aged 65–75 years; healthy older adults	30 vs. 30	18/30 (60.0%) vs. 18/30 (60.0%)	69.0 ± 3.0 vs. 69.0 ± 3.0	22.4 ± 2.6 vs. 22.6 ± 3.6	12 weeks	NMN 250 mg/day vs. placebo	[34]
Qiu 2023 (China)	RCT, OL, LC	Adults with mild essential hypertension; hypertension/metabolic risk	9 vs. 10	4/9 (44.4%) vs. 5/10 (50.0%)	46.00 ± 12.86 vs. 46.70 ± 11.19	22.91 ± 3.41 vs. 22.92 ± 1.86	6 weeks	NMN 800 mg/day + lifestyle modification vs. lifestyle modification alone	[28]
Yoshino 2021 (USA)	RCT, DB, PC	Overweight or obese postmenopausal women with prediabetes; prediabetes/overweight or obesity	13 vs. 12	0/13 (0.0%) vs. 0/12 (0.0%)	62.0 ± 4.0 vs. 61.0 ± 5.0	33.7 ± 1.4 vs. 33.4 ± 1.0	10 weeks	NMN 250 mg/day vs. placebo	[26]
Pencina & Lavu 2023 (USA)	RCT, DB, PC	Overweight or obese middle-aged and older adults; overweight/obesity	12 vs. 8	Overall trial: 16/32 (50.0%)	Overall trial: 63.9 ± 6.1	Overall trial: 29.1 ± 2.9	14 days	MIB-626 1000 mg once daily vs. placebo	[24]
Pencina & Lavu 2023 (USA)	RCT, DB, PC	Overweight or obese middle-aged and older adults; overweight/obesity	12 vs. 8	Overall trial: 16/32 (50.0%)	Overall trial: 63.9 ± 6.1	Overall trial: 29.1 ± 2.9	14 days	MIB-626 1000 mg twice daily vs. placebo	[24]
Okabe 2022 (Japan)	RCT, DB, PC	Healthy adults aged 20–65 years; healthy adults	15 vs. 15	4/15 (26.7%) vs. 4/15 (26.7%)	42.9 ± 12.0 vs. 43.9 ± 9.9	21.3 ± 2.5 vs. 21.1 ± 2.1	12 weeks	NMN 250 mg/day vs. placebo	[22]
Fukamizu 2022 (Japan)	RCT, DB, PC	Healthy adults aged 20–65 years; healthy adults	16 vs. 15	7/16 (43.8%) vs. 7/15 (46.7%)	35.1 ± 7.0 vs. 35.7 ± 7.2	22.9 ± 2.7 vs. 22.1 ± 3.3	4 weeks	NMN 1250 mg/day vs. placebo	[29]
Huang 2022 (China)	RCT, DB, PC	Healthy middle-aged and older adults aged 40–65 years; healthy adults	31 vs. 31	13/31 (41.9%) vs. 15/31 (48.4%)	47.76 ± 6.60 vs. 47.21 ± 6.55	25.26 ± 2.34 vs. 24.72 ± 2.40	60 days	NMN 300 mg/day vs. placebo	[30]
Yi 2023 (India)	RCT, DB, PC	Healthy middle-aged adults aged 40–65 years; healthy adults	20 vs. 20	10/20 (50.0%) vs. 8/20 (40.0%)	51.2 ± 7.0 vs. 46.5 ± 6.7	27.4 ± 4.8 vs. 26.9 ± 4.9	60 days	NMN 300 mg/day vs. placebo	[33]
Yi 2023 (India)	RCT, DB, PC	Healthy middle-aged adults aged 40–65 years; healthy adults	20 vs. 20	6/20 (30.0%) vs. 8/20 (40.0%)	49.5 ± 6.7 vs. 46.5 ± 6.7	27.1 ± 3.9 vs. 26.9 ± 4.9	60 days	NMN 600 mg/day vs. placebo	[33]
Yi 2023 (India)	RCT, DB, PC	Healthy middle-aged adults aged 40–65 years; healthy adults	20 vs. 20	9/20 (45.0%) vs. 8/20 (40.0%)	49.9 ± 6.3 vs. 46.5 ± 6.7	26.9 ± 4.9 vs. 26.9 ± 4.9	60 days	NMN 900 mg/day vs. placebo	[33]
Igarashi 2022 (Japan)	RCT, DB, PC	Healthy older men aged ≥65 years; healthy older adults	21 vs. 21	21/21 (100.0%) vs. 21/21 (100.0%)	71.1 ± 3.9 vs. 71.8 ± 6.1	24.1 ± 1.4 vs. 24.5 ± 1.4	12 weeks	NMN 250 mg/day vs. placebo	[31]
Pencina & Leaf 2025 (USA)	RCT, DB, PC	Hospitalized adults with COVID-19 and AKI; acute illness/COVID-19 with AKI	25 vs. 17	12/25 (48.0%) vs. 11/17 (64.7%)	70.7 ± 14.0 vs. 65.0 ± 12.2	30.0 ± 7.1 vs. 29.7 ± 6.3	14 days	MIB-626 2000 mg/day vs. placebo	[50]
Nakajima 2025 (Japan)	RCT, DB, PC	Healthy adults aged 20–64 years; healthy adults	10 vs. 10	4/10(40.0%) vs. 5/10 (50.0%)	46.9 ± 7.7 vs. 49.7 ± 7.8	22.3 ± 2.7 vs. 22.7 ± 2.2	4 weeks	NMN 750 mg/day vs. placebo	[35]
Nakajima 2025 (Japan)	RCT, DB, PC	Healthy adults aged 20–64 years; healthy adults	10 vs. 10	4/10(40.0%) vs. 5/10 (50.0%)	48.9 ± 8.8 vs. 49.7 ± 7.8	22.7 ± 4.3 vs. 22.7 ± 2.2	4 weeks	NMN 1500 mg/day vs. placebo	[35]

Abbreviations: AKI, acute kidney injury; BID, twice daily; BMI, body mass index; DB, double-blind; NMN, nicotinamide mononucleotide; NR, not reported; PC, placebo-controlled; QD, once daily; RCT, randomized controlled trial; T2DM, type 2 diabetes mellitus. For multi-arm trials, dose-specific intervention groups are displayed separately for descriptive clarity. Shared control groups were handled analytically as described in the Section 2.4. Note: Some RCTs included multiple NMN dose groups. Each row represents a dose-specific comparison with the control group. The number of rows (comparisons) exceeds the number of independent RCTs.

**Table 3:** Summary of pooled safety outcomes for oral NMN or NMN-related supplementation.

Adverse Event Type	Studies Reporting the Outcome, n	Participants, NMN/Control	NMN Events/Total	Control Events/Total	Effect Size (95% CI)	*p* Value	Reference(s)
Any adverse event	10	230/153	58/230	38/153	RD = −0.008 (−0.061, 0.045)	0.761	[22,23,24,25,26,29,30,32,33,50]
Serious adverse events †	10	230/153	7/230	7/153	RD = −0.003 (−0.036, 0.031)/Peto OR = 0.61 (0.18, 2.14)	0.878/0.443	[22,23,24,25,26,29,30,32,33,50]
Withdrawals due to adverse events	9	205/136	2/205	2/136	RD = −0.001 (−0.036, 0.034)	0.950	[22,23,24,25,26,29,30,32,33]
Gastrointestinal adverse events	7	168/92	16/168	17/92	RD = −0.047 (−0.121, 0.027)	0.210	[22,24,25,29,32,33,50]
Nervous system adverse events	5	145/69	11/145	7/69	RD = −0.026 (−0.117, 0.065)	0.581	[22,24,25,33,50]
Skin/allergic adverse events	5	122/66	7/122	2/66	RD = 0.023 (−0.053, 0.098)	0.560	[22,24,29,32,33]
Other adverse events	8	199/123	26/199	11/123	RD = 0.017 (−0.041, 0.075)	0.562	[22,24,25,29,30,32,33,50]

Abbreviations: NMN, nicotinamide mononucleotide; RD, risk difference; OR, odds ratio; CI, confidence interval. Note: † Because event rates were low and events were few across trials, we pooled serious adverse events using the Peto odds ratio. For other binary safety outcomes, we used risk differences. Ten studies contributed numbers to the serious adverse event analysis, but only two studies reported at least one such event. A negative RD means the NMN group had a lower event rate. All confidence intervals crossed the null value. This indicates no statistically significant difference between the groups.

## Data Availability

All data generated or analyzed during this study are included in this article and its Supplementary Materials. The analytic code is available from the corresponding author upon reasonable request.

## Supplementary Materials

The following supporting information can be downloaded at: https://www.mdpi.com/article/10.3390/nu18142251/s1, Table S1: Complete search strategies for PubMed/MEDLINE, Embase, Scopus, Web of Science, CNKI, and Wanfang; Figure S1: Forest plot of gastrointestinal adverse events; Figure S2: Forest plot of nervous system adverse events; Figure S3: Forest plot of skin/allergic adverse events; Figure S4: Forest plot of other adverse events; Figure S5: Forest plot of withdrawals due to adverse events; Figure S6: Forest plot of body mass index (BMI); Figure S7: Forest plot of glycated hemoglobin (HbA1c); Figure S8: Forest plot of high-density lipoprotein cholesterol (HDL-C); Figure S9: Forest plot of low-density lipoprotein cholesterol (LDL-C); Figure S10: Forest plot of total cholesterol (TC); Figure S11: Forest plot of triglycerides (TG); Figure S12: Funnel plot of alanine aminotransferase (ALT); Figure S13: Trim-and-fill funnel plot for ALT; Figure S14: Funnel plots for outcomes with at least ten comparisons; Figure S15: Forest plots of exploratory subgroup analyses for the effects of NMN supplementation on blood pressure; Table S2: GRADE evidence profiles for prespecified clinical and safety outcomes; Table S3: Detailed results of sensitivity analyses; Table S4: PRISMA 2020 checklist; Table S5: Full-text reports excluded after eligibility assessment and reasons for exclusion; Table S6. Exploratory subgroup analyses for the effects of NMN supplementation on systolic and diastolic blood pressure.

## Abbreviations

The following abbreviations are used in this manuscript:

Abbreviation	Definition
AE	adverse event
AKI	acute kidney injury
ALT	alanine aminotransferase
AST	aspartate aminotransferase
BID	twice daily
BMI	body mass index
CI	confidence interval
DB	double blind
DBP	diastolic blood pressure
EFSA	European Food Safety Authority
FPG	fasting plasma glucose
GRADE	Grading of Recommendations Assessment, Development and Evaluation
HbA1c	glycated hemoglobin
HDL-C	high-density lipoprotein cholesterol
HOMA-IR	homeostatic model assessment of insulin resistance
LDL-C	low-density lipoprotein cholesterol
MIB-626	microcrystalline β-nicotinamide mononucleotide formulation
NAD^+^	nicotinamide adenine dinucleotide
NMN	nicotinamide mononucleotide
NR	not reported
OR	odds ratio
PC	Placebo controlled
PRISMA	Preferred Reporting Items for Systematic Reviews and Meta-Analyses
QD	once daily
RCT	randomized controlled trial
RD	risk difference
RoB	risk of bias
SAE	serious adverse event
SBP	systolic blood pressure
SMD	standardized mean difference
T2DM	type 2 diabetes mellitus
TC	total cholesterol
TG	triglyceride
