# Supplementary material for: Safety and Metabolism-Related Outcomes of Oral Nicotinamide Mononucleotide Supplementation in Adults: A Systematic Review and Meta-Analysis
Source: Nutrients. 2026 Jul 10;18(14):2251. doi: 10.3390/nu18142251 (PMC13414721; doi:10.3390/nu18142251)
Supplement: Supplementary file 1 [file nutrients-18-02251-s001.zip › nutrients-4378122-supplementary.pdf]

Supplementary Materials

Safety and Metabolism-Related Outcomes of Oral Nicotinamide Mononucleotide Supplementation in Adults: A Systematic Review and Meta-Analysis

Supplementary Table S1. Complete search strategies for PubMed/MEDLINE, Embase, Scopus, Web of Science, CNKI, and Wanfang.

Database	Search terms
PubMed/MEDLINE （72）	(("Nicotinamide Mononucleotide"[Mesh] OR "nicotinamide mononucleotide"[Title/Abstract] OR "beta-nicotinamide mononucleotide"[Title/Abstract] OR "β-nicotinamide mononucleotide"[Title/Abstract] OR "NMN"[Title/Abstract])<br>AND<br>("Randomized Controlled Trial"[Publication Type] OR "Controlled Clinical Trial"[Publication Type] OR randomized[Title/Abstract] OR randomised[Title/Abstract] OR randomly[Title/Abstract] OR placebo[Title/Abstract] OR "clinical trial"[Title/Abstract] OR trial[Title/Abstract]))<br>NOT<br>(animals[MeSH Terms] NOT humans[MeSH Terms])
Embase （146）	('nicotinamide mononucleotide'/exp OR 'nicotinamide mononucleotide':ti,ab,kw OR 'beta nicotinamide mononucleotide':ti,ab,kw OR 'β-nicotinamide mononucleotide':ti,ab,kw OR nmn:ti,ab,kw)<br>AND<br>('randomized controlled trial'/exp OR 'controlled clinical trial'/exp OR random*:ti,ab,kw OR placebo*:ti,ab,kw OR 'clinical trial':ti,ab,kw OR trial:ti,ab,kw)<br>AND [humans]/lim<br>NOT ([animals]/lim NOT [humans]/lim)
Scopus （230）	TITLE-ABS-KEY("nicotinamide mononucleotide" OR "beta-nicotinamide mononucleotide" OR "β-nicotinamide mononucleotide" OR NMN)<br>AND<br>TITLE-ABS-KEY(random* OR placebo* OR "randomized controlled trial" OR "randomised controlled trial" OR "controlled clinical trial" OR "clinical trial" OR trial)
Web of Science （164）	("nicotinamide mononucleotide" OR "beta-nicotinamide mononucleotide" OR "β-nicotinamide mononucleotide" OR NMN)<br>AND<br>(random* OR placebo* OR "randomized controlled trial" OR "randomised controlled trial" OR "controlled clinical trial" OR "clinical trial" OR trial)
CNKI （0）	("烟酰胺单核苷酸" OR "尼克酰胺单核苷酸" OR "NMN")<br>AND<br>("随机" OR "随机对照" OR "随机对照试验" OR "临床试验" OR "安慰剂" OR "双盲" OR "对照")
Wanfang （262）	主题:("烟酰胺单核苷酸" OR "β-烟酰胺单核苷酸" OR "尼克酰胺单核苷酸" OR "NMN")<br>AND<br>主题:("随机" OR "随机对照" OR "随机对照试验" OR "临床试验" OR "安慰剂" OR "双盲" OR "对照")

Note: Chinese search terms were used only for CNKI and Wanfang searches to ensure database-specific retrieval. The Chinese terms “烟酰胺单核苷酸”, “β-烟酰胺单核苷酸”, and “尼克酰胺单核苷酸” refer to nicotinamide mononucleotide or β-nicotinamide mononucleotide. The Chinese terms “随机”, “随机对照”, “随机对照试验”, “临床试验”, “安慰剂”, “双盲”, and “对照” refer to randomized, randomized controlled, randomized controlled trial, clinical trial, placebo, double-blind, and controlled, respectively.

Supplementary Figure S1. Forest plot of gastrointestinal adverse events.

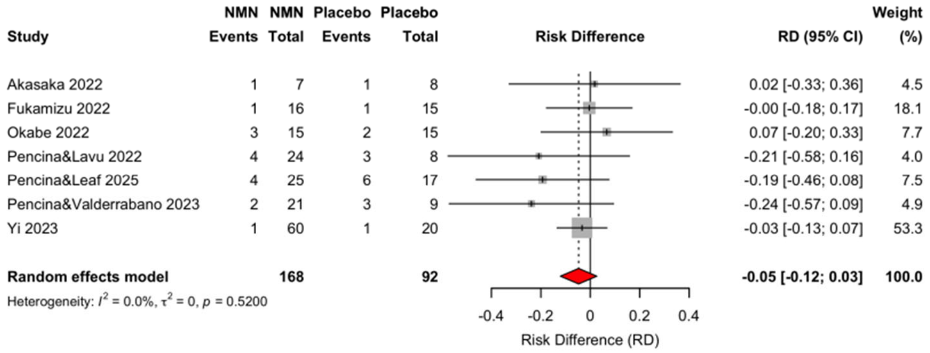

Supplementary Figure S2. Forest plot of nervous system adverse events.

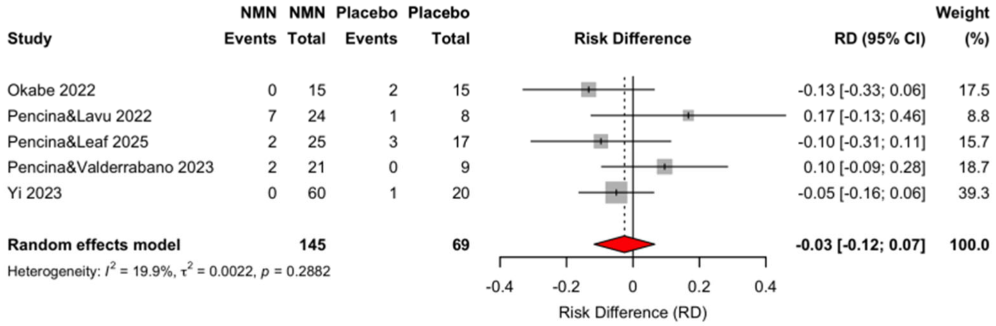

Supplementary Figure S3. Forest plot of skin/allergic adverse events.

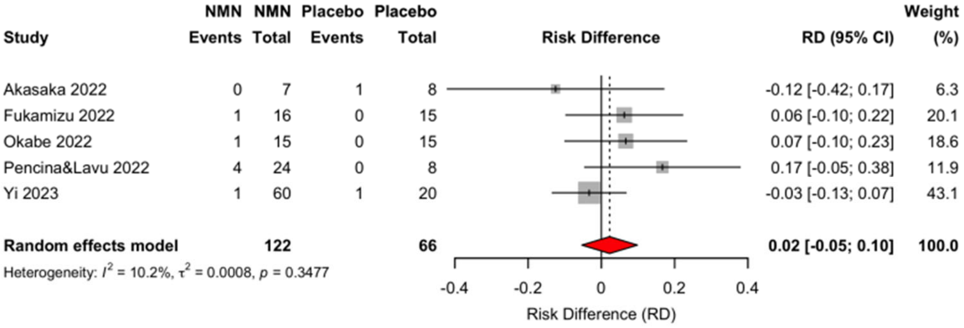

Supplementary Figure S4. Forest plot of other adverse events.

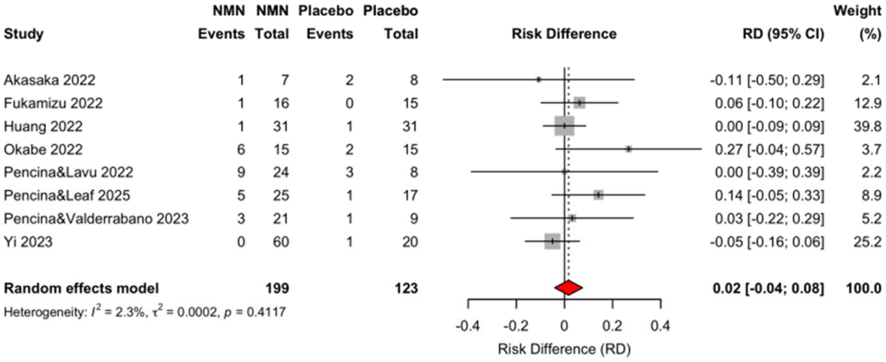

Supplementary Figure S5. Forest plot of withdrawals due to adverse events.

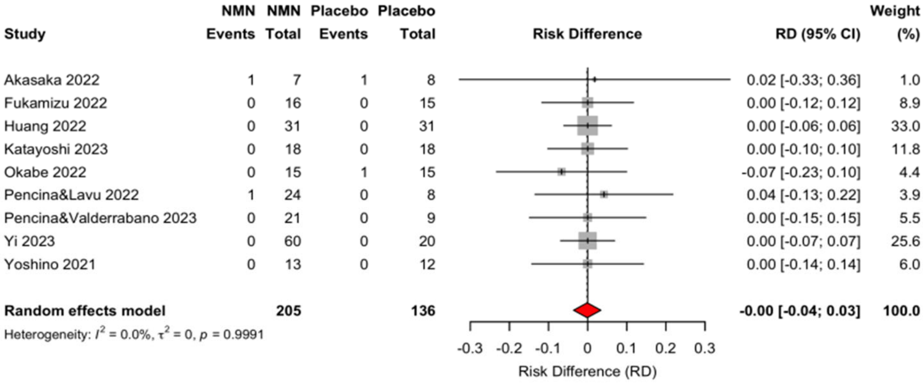

Supplementary Figure S6. Forest plot of body mass index (BMI).

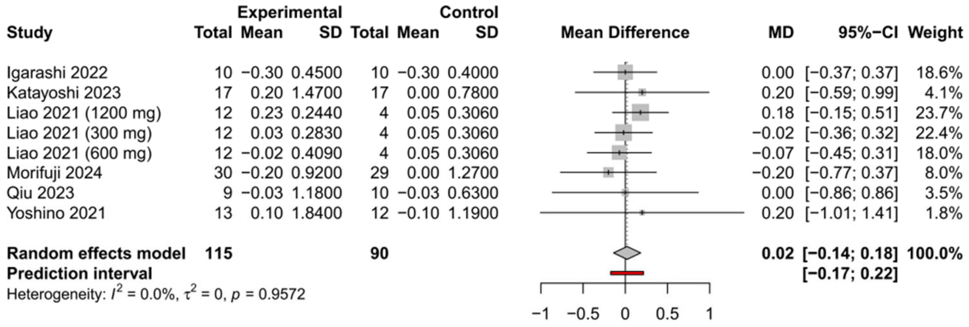

Supplementary Figure S7. Forest plot of glycated hemoglobin (HbA1c).

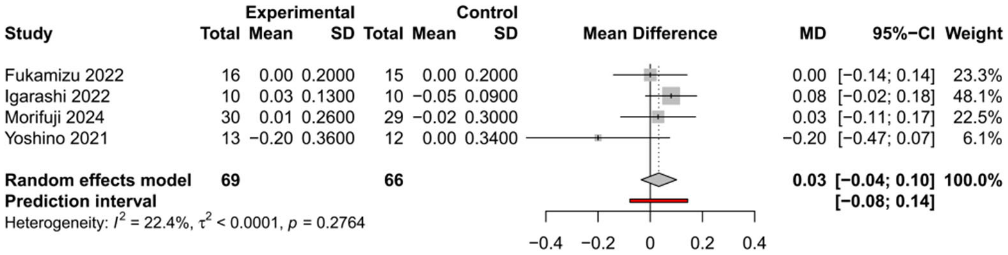

Supplementary Figure S8. Forest plot of high-density lipoprotein cholesterol (HDL-C).

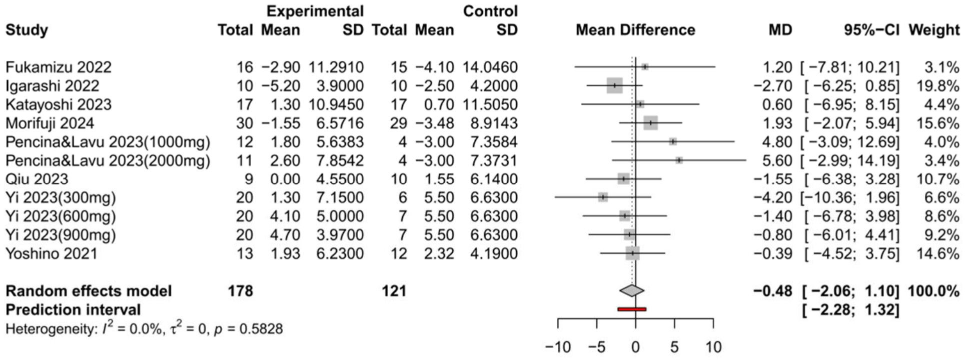

Supplementary Figure S9. Forest plot of low-density lipoprotein cholesterol (LDL-C).

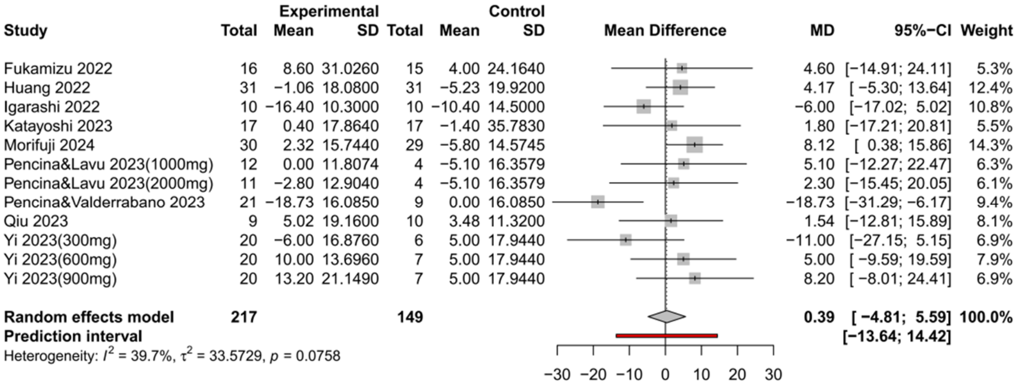

Supplementary Figure S10. Forest plot of total cholesterol (TC).

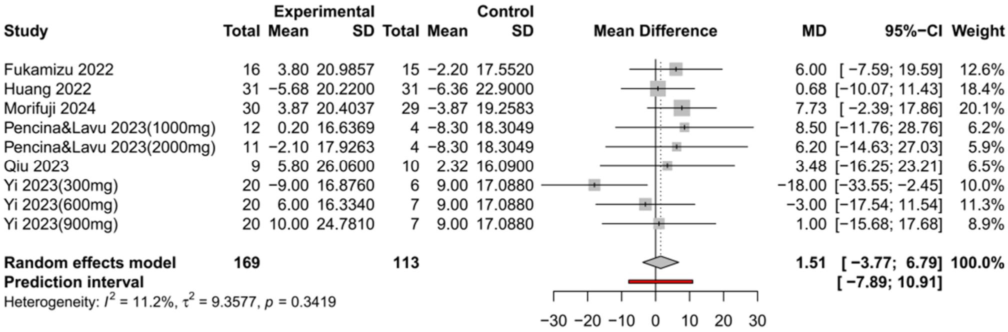

Supplementary Figure S11. Forest plot of triglycerides (TG).

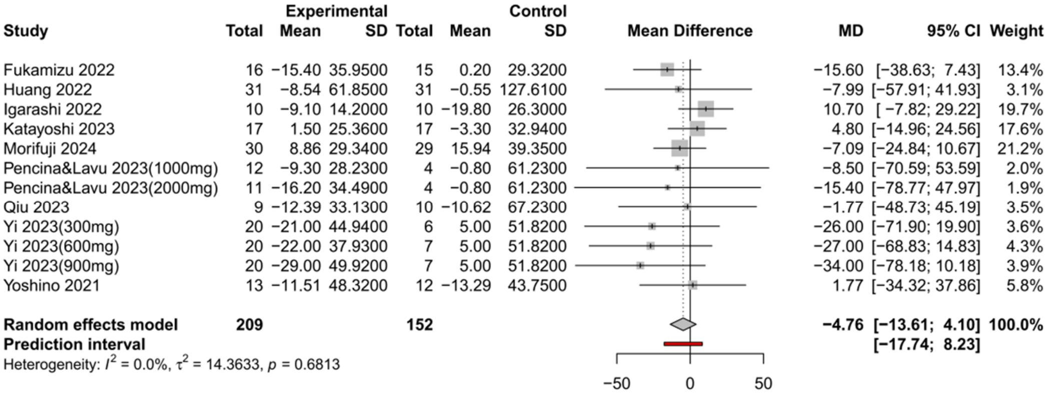

Supplementary Figure S12. Funnel plot of alanine aminotransferase (ALT).

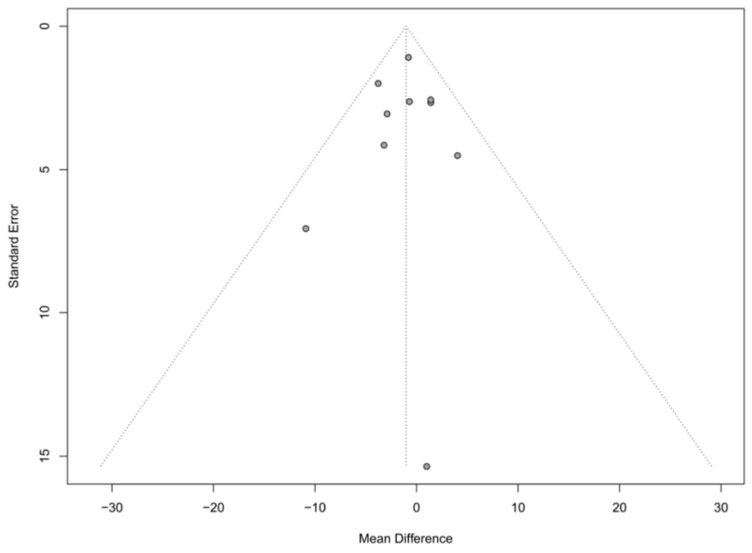

Supplementary Figure S13. Trim-and-fill funnel plot for ALT.

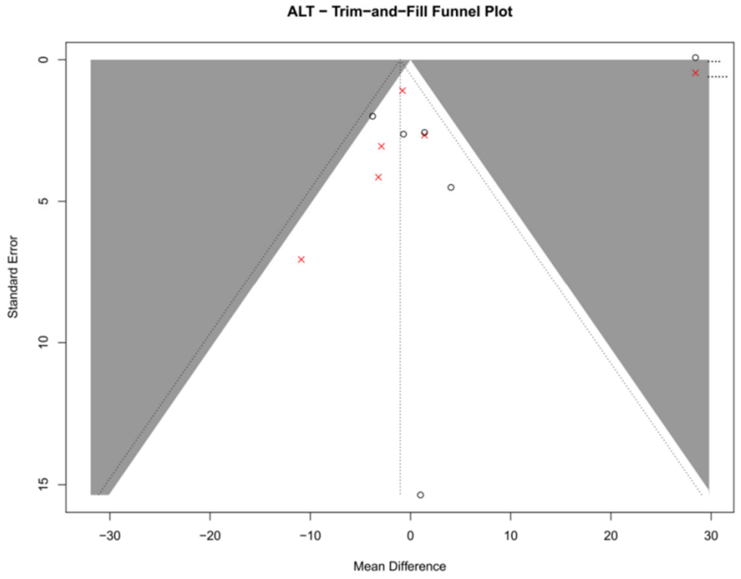

Supplementary Figure S14. Funnel plots for outcomes with at least ten comparisons (AST, HDL-C, LDL-C, TG, SBP, DBP, and total adverse events).

(a) AST

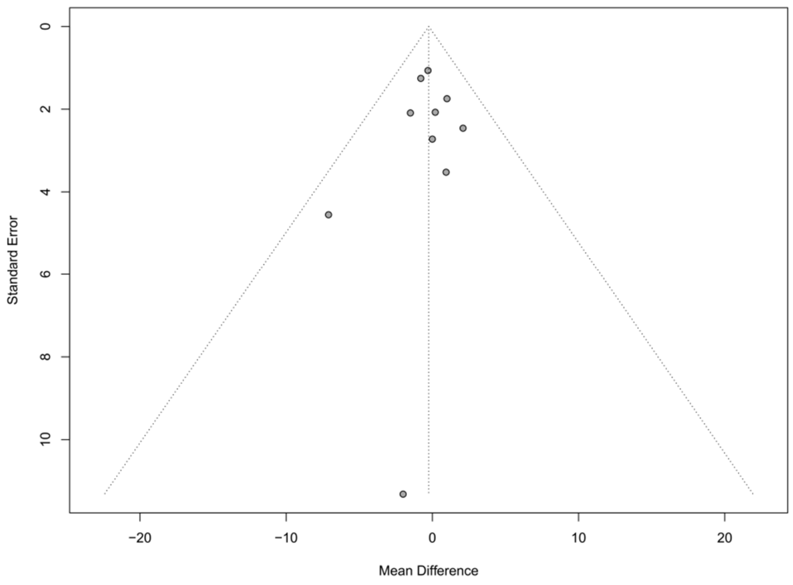

0
2
4
6
8
10
Standard Error
−20
−10
0
10
20
Mean Difference

(b)HDL-C

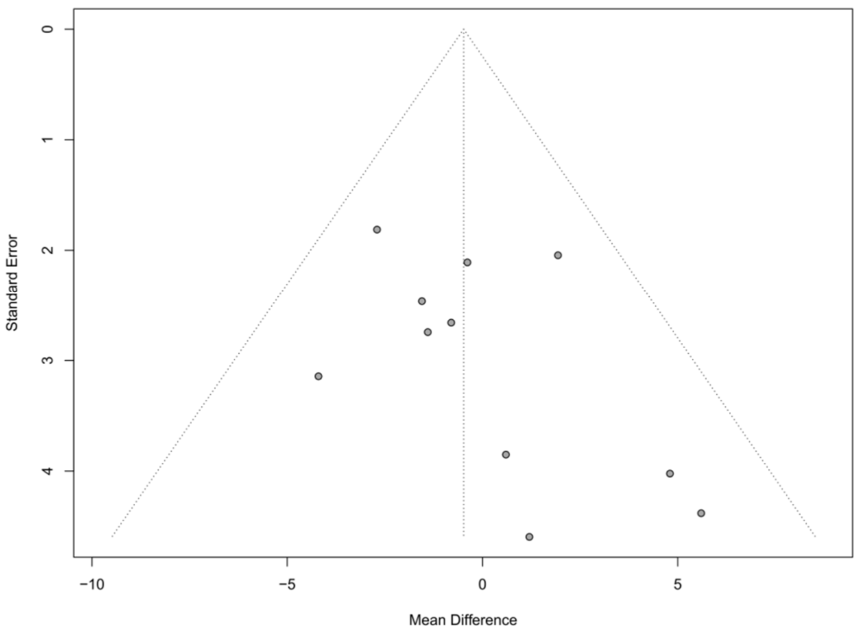

0
1
2
3
4
Standard Error
−10
−5
0
5
Mean Difference

(c)LDL-C

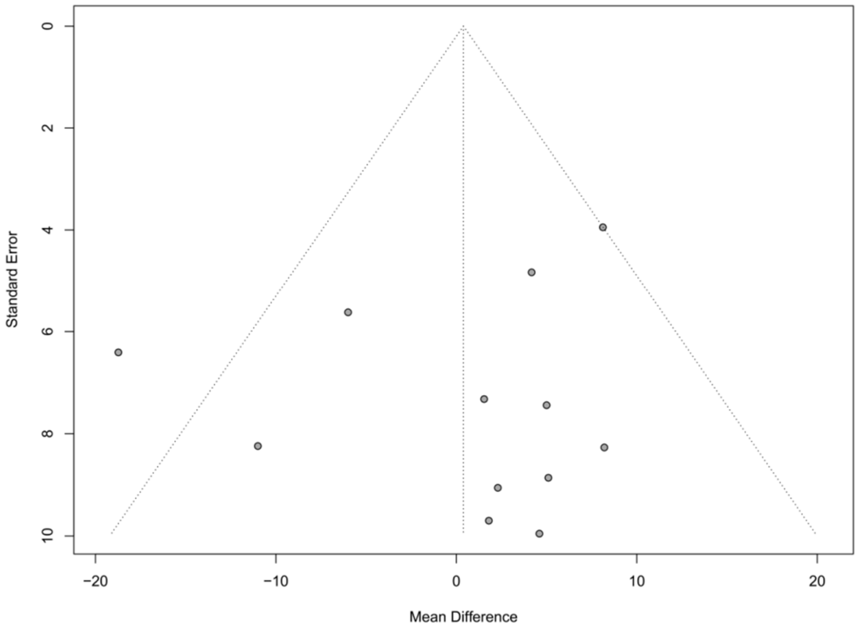

(d)TG

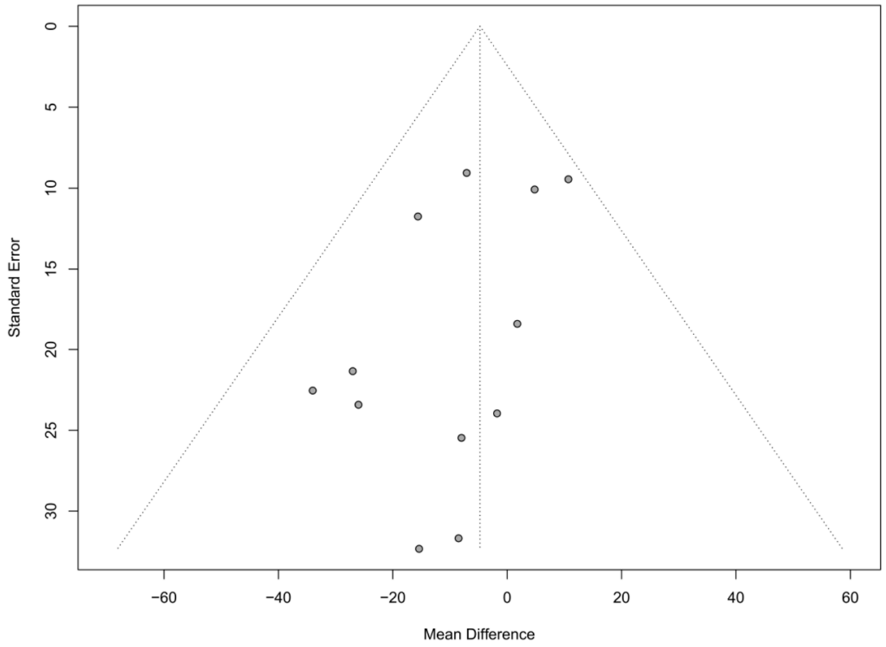

Standard Error
Mean Difference

(e)SBP

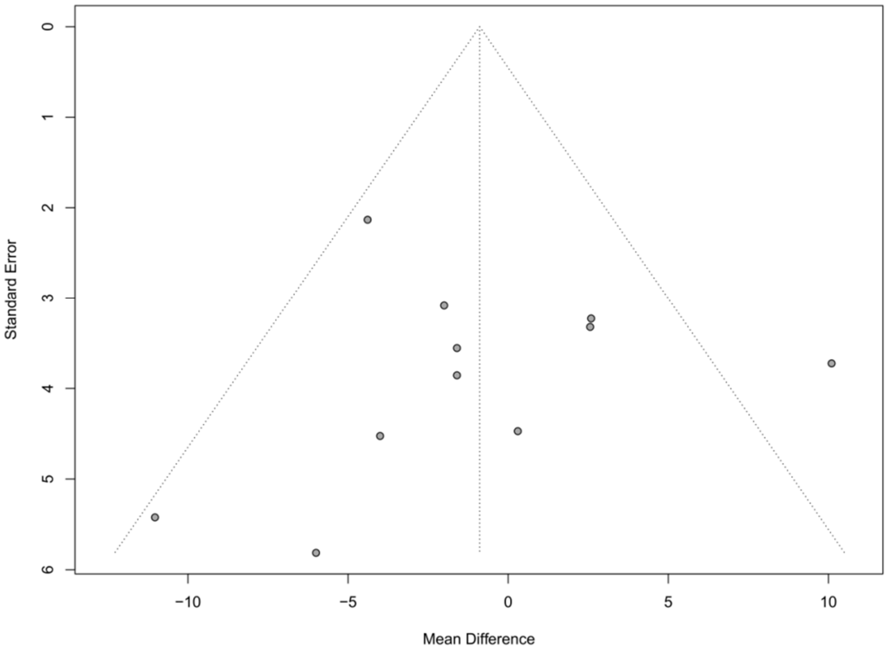

Standard Error
Mean Difference

(f)DBP

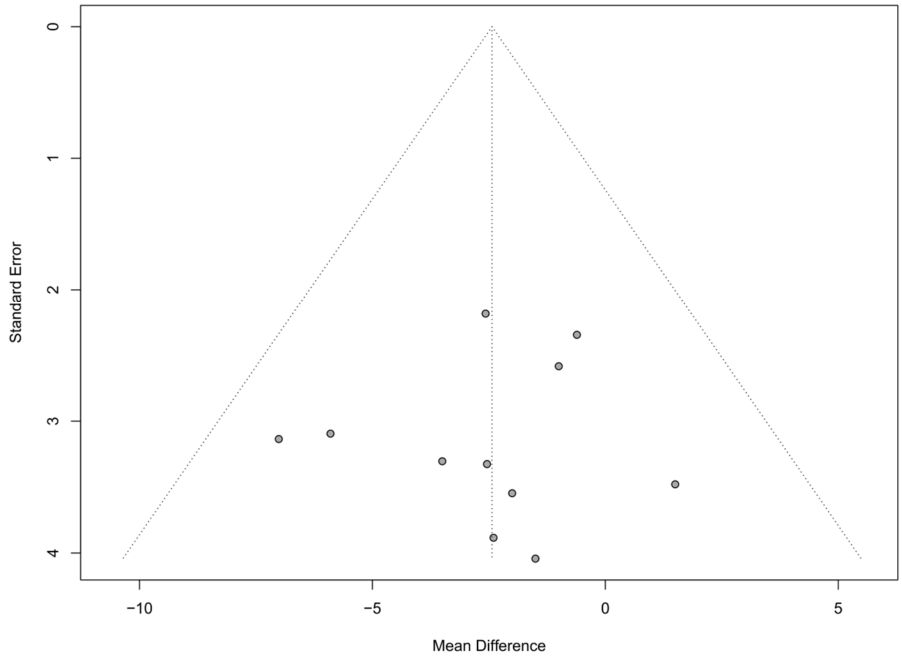

(g) Total adverse events

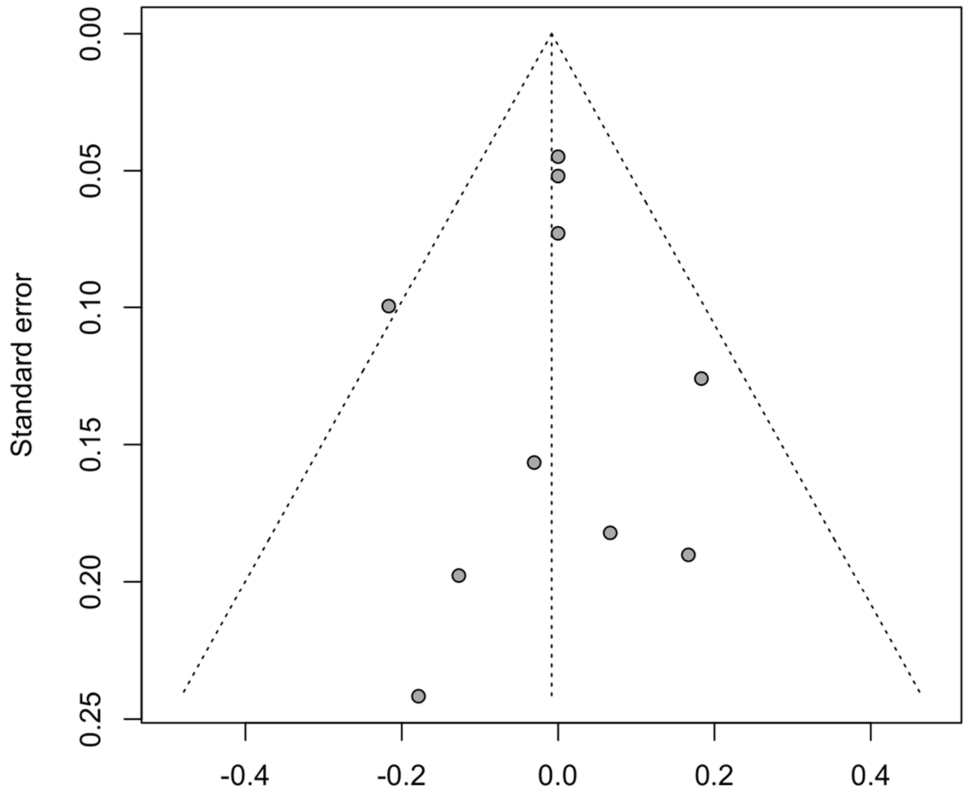

Supplementary Figure S15. Forest plots of exploratory subgroup analyses for the effects of

NMN supplementation on blood pressure. (A) DBP stratified by age group; (B) DBP stratified by metabolic status; (C) DBP stratified by intervention duration; (D) SBP stratified by age group; (E) SBP stratified by metabolic status; and (F) SBP stratified by intervention duration. Detailed subgroup-specific estimates and P values for subgroup differences are summarized in Supplementary Table S6.

(A)

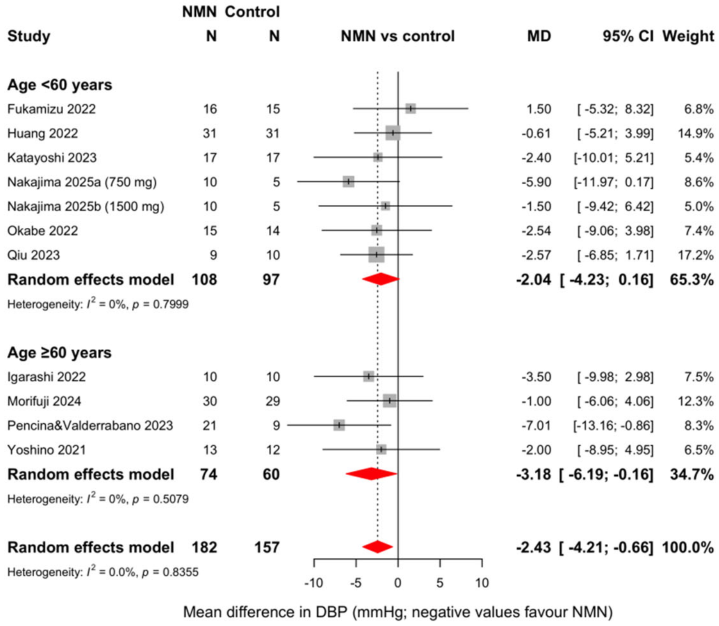

(B)

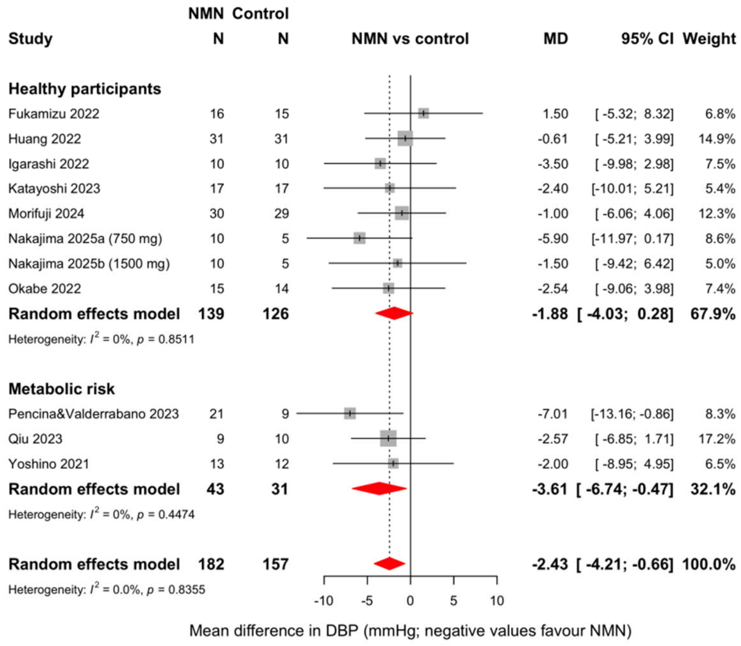

（C）

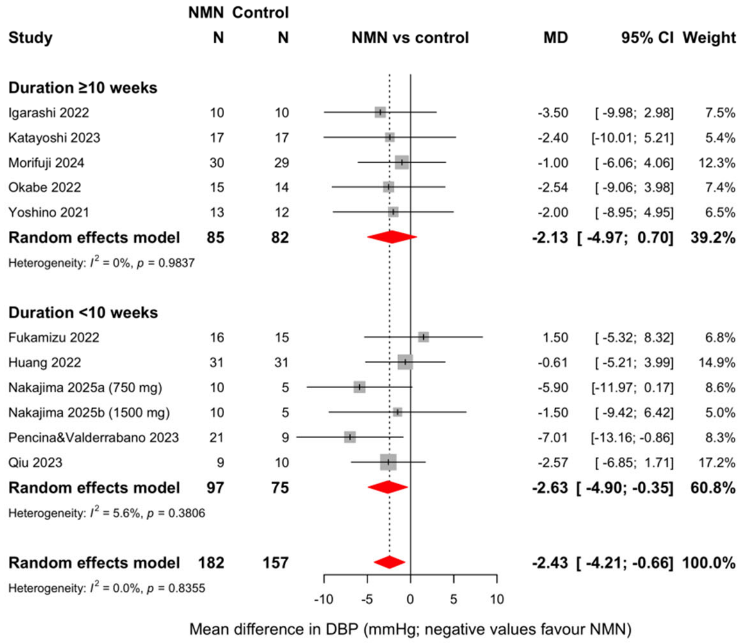

Study
NMN N
Control N
NMN vs control
MD
95% CI
Weight
Duration ≥10 weeks
Igarashi 2022 10 10 -3.50 [-9.98; 2.98] 7.5%
Katayoshi 2023 17 17 -2.40 [-10.01; 5.21] 5.4%
Morifuji 2024 30 29 -1.00 [-6.06; 4.06] 12.3%
Okabe 2022 15 14 -2.54 [-9.06; 3.98] 7.4%
Yoshino 2021 13 12 -2.00 [-8.95; 4.95] 6.5%
Random effects model 85 82 -2.13 [-4.97; 0.70] 39.2%
Heterogeneity: I^2 = 0%, p = 0.9837
Duration <10 weeks
Fukamizu 2022 16 15 1.50 [-5.32; 8.32] 6.8%
Huang 2022 31 31 -0.61 [-5.21; 3.99] 14.9%
Nakajima 2025a (750 mg) 10 5 -5.90 [-11.97; 0.17] 8.6%
Nakajima 2025b (1500 mg) 10 5 -1.50 [-9.42; 6.42] 5.0%
Pencina&Valderrabano 2023 21 9 -7.01 [-13.16; -0.86] 8.3%
Qiu 2023 9 10 -2.57 [-6.85; 1.71] 17.2%
Random effects model 97 75 -2.63 [-4.90; -0.35] 60.8%
Heterogeneity: I^2 = 5.6%, p = 0.3806
Random effects model 182 157 -2.43 [-4.21; -0.66] 100.0%
Heterogeneity: I^2 = 0.0%, p = 0.8355
-10 -5 0 5 10
Mean difference in DBP (mmHg; negative values favour NMN)

（D）

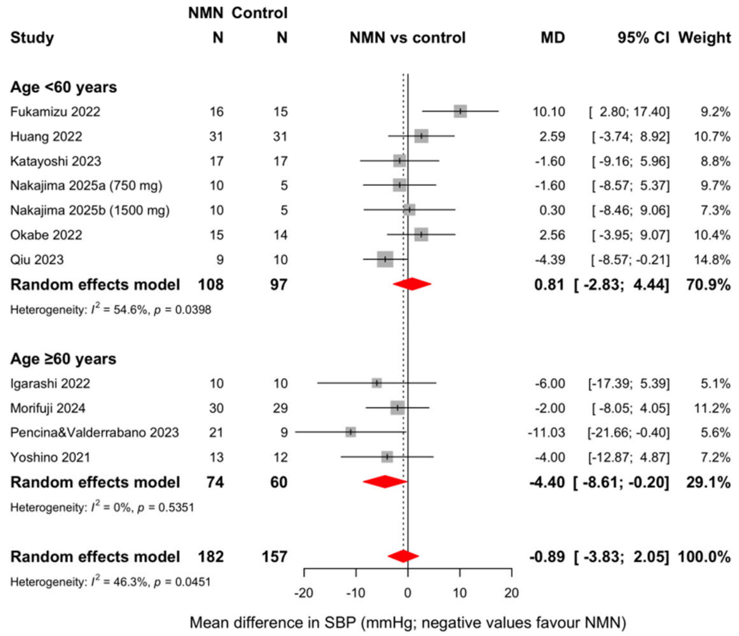

Study
NMN N
Control N
NMN vs control
MD
95% CI
Weight
Age <60 years
Fukamizu 2022 16 15 10.10 [2.80; 17.40] 9.2%
Huang 2022 31 31 2.59 [-3.74; 8.92] 10.7%
Katayoshi 2023 17 17 -1.60 [-9.16; 5.96] 8.8%
Nakajima 2025a (750 mg) 10 5 -1.60 [-8.57; 5.37] 9.7%
Nakajima 2025b (1500 mg) 10 5 0.30 [-8.46; 9.06] 7.3%
Okabe 2022 15 14 2.56 [-3.95; 9.07] 10.4%
Qiu 2023 9 10 -4.39 [-8.57; -0.21] 14.8%
Random effects model 108 97 0.81 [-2.83; 4.44] 70.9%
Heterogeneity: I^2 = 54.6%, p = 0.0398
Age ≥60 years
Igarashi 2022 10 10 -6.00 [-17.39; 5.39] 5.1%
Morifuji 2024 30 29 -2.00 [-8.05; 4.05] 11.2%
Pencina&Valderrabano 2023 21 9 -11.03 [-21.66; -0.40] 5.6%
Yoshino 2021 13 12 -4.00 [-12.87; 4.87] 7.2%
Random effects model 74 60 -4.40 [-8.61; -0.20] 29.1%
Heterogeneity: I^2 = 0%, p = 0.5351
Random effects model 182 157 -0.89 [-3.83; 2.05] 100.0%
Heterogeneity: I^2 = 46.3%, p = 0.0451
-20 -10 0 10 20
Mean difference in SBP (mmHg; negative values favour NMN)

（E）

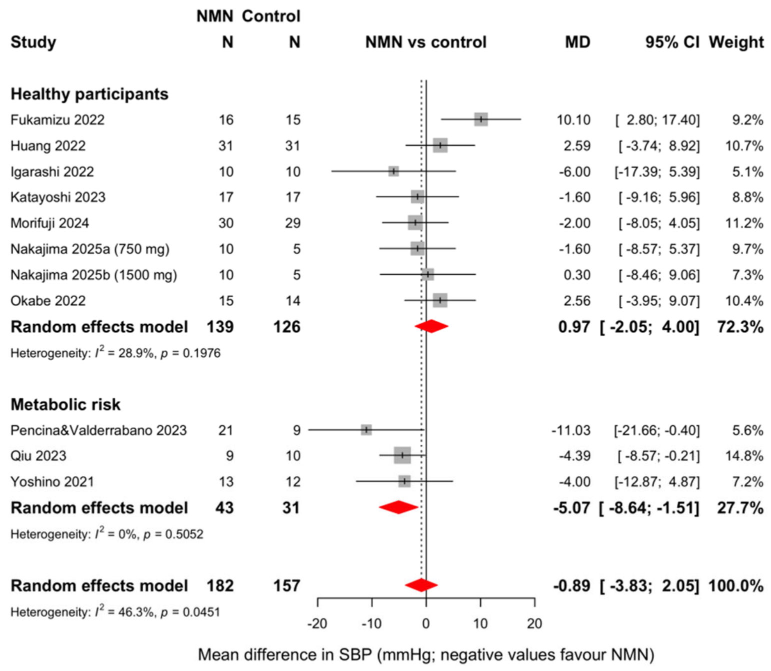
NMN Control
Study N N NMN vs control MD 95% CI Weight
Healthy participants
Fukamizu 2022 16 15 10.10 [2.80; 17.40] 9.2%
Huang 2022 31 31 2.59 [-3.74; 8.92] 10.7%
Igarashi 2022 10 10 -6.00 [-17.39; 5.39] 5.1%
Katayoshi 2023 17 17 -1.60 [-9.16; 5.96] 8.8%
Morifuji 2024 30 29 -2.00 [-8.05; 4.05] 11.2%
Nakajima 2025a (750 mg) 10 5 -1.60 [-8.57; 5.37] 9.7%
Nakajima 2025b (1500 mg) 10 5 0.30 [-8.46; 9.06] 7.3%
Okabe 2022 15 14 2.56 [-3.95; 9.07] 10.4%
Random effects model 139 126 0.97 [-2.05; 4.00] 72.3%
Heterogeneity: I^2 = 28.9%, p = 0.1976
Metabolic risk
Pencina&Valderrabano 2023 21 9 -11.03 [-21.66; -0.40] 5.6%
Qiu 2023 9 10 -4.39 [-8.57; -0.21] 14.8%
Yoshino 2021 13 12 -4.00 [-12.87; 4.87] 7.2%
Random effects model 43 31 -5.07 [-8.64; -1.51] 27.7%
Heterogeneity: I^2 = 0%, p = 0.5052
Random effects model 182 157 -0.89 [-3.83; 2.05] 100.0%
Heterogeneity: I^2 = 46.3%, p = 0.0451
-20 -10 0 10 20
Mean difference in SBP (mmHg; negative values favour NMN)

(F)

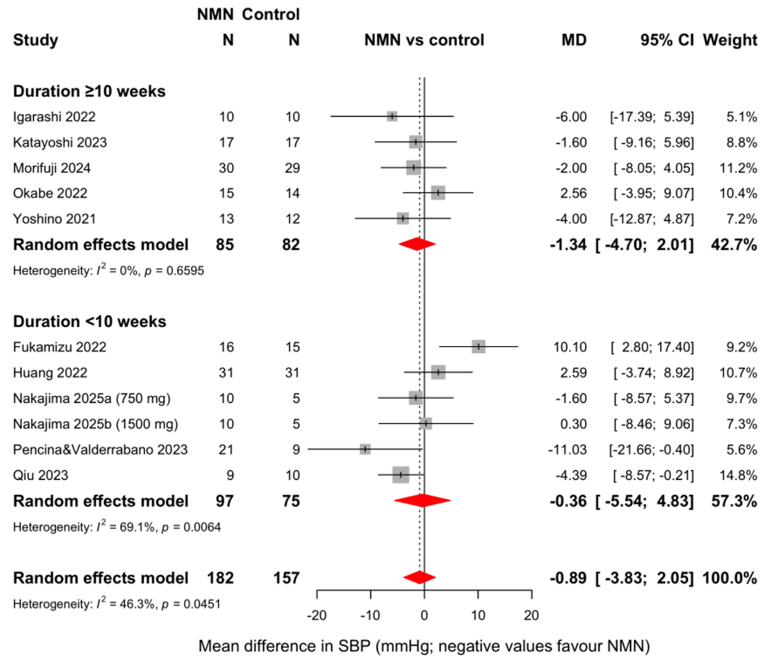
NMN Control
Study N N NMN vs control MD 95% CI Weight
Duration ≥10 weeks
Igarashi 2022 10 10 -6.00 [-17.39; 5.39] 5.1%
Katayoshi 2023 17 17 -1.60 [-9.16; 5.96] 8.8%
Morifuji 2024 30 29 -2.00 [-8.05; 4.05] 11.2%
Okabe 2022 15 14 2.56 [-3.95; 9.07] 10.4%
Yoshino 2021 13 12 -4.00 [-12.87; 4.87] 7.2%
Random effects model 85 82 -1.34 [-4.70; 2.01] 42.7%
Heterogeneity: I^2 = 0%, p = 0.6595
Duration <10 weeks
Fukamizu 2022 16 15 10.10 [2.80; 17.40] 9.2%
Huang 2022 31 31 2.59 [-3.74; 8.92] 10.7%
Nakajima 2025a (750 mg) 10 5 -1.60 [-8.57; 5.37] 9.7%
Nakajima 2025b (1500 mg) 10 5 0.30 [-8.46; 9.06] 7.3%
Pencina&Valderrabano 2023 21 9 -11.03 [-21.66; -0.40] 5.6%
Qiu 2023 9 10 -4.39 [-8.57; -0.21] 14.8%
Random effects model 97 75 -0.36 [-5.54; 4.83] 57.3%
Heterogeneity: I^2 = 69.1%, p = 0.0064
Random effects model 182 157 -0.89 [-3.83; 2.05] 100.0%
Heterogeneity: I^2 = 46.3%, p = 0.0451
-20 -10 0 10 20
Mean difference in SBP (mmHg; negative values favour NMN)

Supplementary Table S2. GRADE evidence profiles for prespecified clinical and safety outcomes.
The GRADE certainty of evidence for the main clinical and safety outcomes is summarized in Supplementary Table S2.

Outcome	Studies/comparisons (participants)	Effect estimate (95% CI)	Heterogeneity	Certainty of evidence	Main reasons for downgrading
ALT	10 comparisons (330)	MD = -1.03 U/L (-2.51 to 0.44)	I^2 = 0%	Moderate	Imprecision
AST	10 comparisons (330)	MD = -0.24 U/L (-1.43 to 0.94)	I^2 = 0%	Moderate	Imprecision
Body weight	8 comparisons (215)	MD = -0.32 kg (-1.19 to 0.54)	I^2 = 14.7%	Moderate	Imprecision
BMI	8 comparisons (205)	MD = 0.02 kg/m² (-0.14 to 0.18)	I^2 = 0%	Moderate	Imprecision
Fasting plasma glucose	7 comparisons (250)	MD = 0.73 mg/dL (-1.36 to 2.82)	I^2 = 0%	Moderate	Imprecision
HbA1c	4 comparisons (135)	MD = 0.03% (-0.04 to 0.10)	I^2 = 22.4%	Low	Imprecision, indirectness
HOMA-IR	5 comparisons (160)	MD = -0.22 (-0.55 to 0.11)	I^2 = 0%	Very low	Serious imprecision, indirectness
LDL-C	12 comparisons (366)	MD = 0.39 mg/dL (-4.81 to 5.59)	I^2 = 39.7%	Low	Inconsistency, imprecision, indirectness
Triglycerides	12 comparisons (361)	MD = -4.76 mg/dL (-13.61 to 4.10)	I^2 = 0%	Moderate	Imprecision
SBP	11 comparisons (339)	MD = -0.89 mmHg (-3.83 to 2.05)	I^2 = 46.3%	Low	Inconsistency, imprecision, indirectness
DBP	11 comparisons (339)	MD = -2.43 mmHg (-4.21 to -0.66)	I^2 = 0%	Low	Imprecision, indirectness

Any adverse event	10 studies (383)	RD = -0.008 (-0.061 to 0.045)	$I^2 = 0\%$	Moderate	Imprecision
Serious adverse events†	10 studies (383)	Peto OR = 0.61 (0.18 to 2.14)	$I^2 = 0\%$	Very low	Very serious imprecision

Abbreviations: ALT, alanine aminotransferase; AST, aspartate aminotransferase; BMI, body mass index; CI, confidence interval; DBP, diastolic blood pressure; FPG, fasting plasma glucose; HbA1c, glycated hemoglobin; HOMA-IR, homeostatic model assessment of insulin resistance; LDL-C, low-density lipoprotein cholesterol; MD, mean difference; OR, odds ratio; RD, risk difference; SBP, systolic blood pressure.

Notes: Certainty of evidence was assessed using the GRADE framework. For serious adverse events, certainty was downgraded to very low because events were sparse and the confidence interval was wide. For DBP, certainty was downgraded to low because of limited sample size, indirectness, and because blood pressure was not the primary endpoint in most trials. For HOMA-IR, certainty was rated as very low because the analysis was based on a limited number of comparisons and should be interpreted as hypothesis-generating.

Supplementary Table S3. Detailed results of sensitivity analyses

Outcome	Main analysis result (95% CI)	Main I^2 (%)	Analyses removed (n)	Sensitivity key finding	I^2 after removal (%)†	Conclusion changed
ALT	MD = -1.03 (-2.51, 0.44)	0	10	Pooled MD ranged -1.87 to -0.32, all CIs crossed 0	0	No
AST	MD = -0.24 (-1.43, 0.94)	0	10	Pooled MD ranged -0.98 to 0.15, all CIs crossed 0	0	No
Body weight	MD = -0.32 (-1.19, 0.54)	14.7	8	MD remained non-significant after any removal	0–25.3	No
BMI	MD = 0.02 (-0.14, 0.18)	0	8	No significant change in any leave-one-out analysis	0	No
Fasting glucose	MD = 0.73 (-1.36, 2.82)	0	7	Pooled MD consistently non-significant	0	No
HDL-C	MD = -0.48 (-2.06, 1.10)	0	11	All leave-one-out analyses maintained null conclusion	0	No
LDL-C	MD = 0.39 (-4.81, 5.59)	39.7	12	After removing Pencina & Valderrabano 2023, I^2=0%, MD remained non-significant	0–43.2	No
TC	MD = 1.51 (-3.77, 6.79)	11.2	9	Removing Yi 2023 multi-arm did not change null result	0–21.5	No
TG	MD = -4.76 (-13.61, 4.10)	0	12	No significant effect in any leave-one-out analysis	0	No
HbA1c	MD = 0.03 (-0.04, 0.10)	22.4	4	Pooled MD ranged from -0.01 to 0.05, and all CIs crossed 0	0-48.2	No
SBP	MD = -0.89 (-3.83, 2.05)	46.3	11	After removing Fukamizu 2022, I^2=0%, MD=-2.00 (-4.24,0.24), still non-significant	0–49.8	No
DBP	MD = -2.43 (-4.21, -0.66); P=0.007	0	11	Pooled MD ranged from -2.75 to -2.02 mmHg; all 95% CIs remained below 0, and all P values were <0.05.	0	No‡

Any adverse event	RD = -0.008 (-0.061, 0.045)	0	10	Pooled RD ranged -0.023 to 0.007, all CIs crossed 0	0	No

Table footnotes:

† I^2 ranges are estimated based on leave-one-out fluctuations; exact values depend on the software used.

‡ DBP remained statistically significant ($P < 0.05$) in every leave-one-out analysis.

RD: risk difference; MD: mean difference; CI: confidence interval. Leave-one-out sensitivity analysis was performed by sequentially removing each study or independent comparison

Supplementary Table S4. PRISMA 2020 checklist.

Section and Topic	Item #	Checklist item	Location where item is reported
**TITLE**			
Title	1	Identify the report as a systematic review.	Title page
ABSTRACT			
Abstract	2	See the PRISMA 2020 for Abstracts checklist.	Abstract
INTRODUCTION			
Rationale	3	Describe the rationale for the review in the context of existing knowledge.	Introduction, paragraphs 1-3
Objectives	4	Provide an explicit statement of the objective(s) or question(s) the review addresses.	Introduction, final paragraph
METHODS			
Eligibility criteria	5	Specify the inclusion and exclusion criteria for the review and how studies were grouped for the syntheses.	Section 2.2 Search strategy and eligibility criteria; Section 2.4 Data extraction, handling of multi-arm studies, and statistical analysis
Information sources	6	Specify all databases, registers, websites, organisations, reference lists and other sources searched or consulted to identify studies. Specify the date when each source was last searched or consulted.	Section 2.2 Search strategy and eligibility criteria; Figure 2; Supplementary Table S1
Search strategy	7	Present the full search strategies for all databases, registers and websites, including any filters and limits used.	Supplementary Table S1
Selection process	8	Specify the methods used to decide whether a study met the inclusion criteria of the review, including how many reviewers screened each record and each report retrieved, whether they worked independently, and if applicable, details of automation tools used in the process.	Section 2.4 Data extraction, handling of multi-arm studies, and statistical analysis; Figure 2
Data collection process	9	Specify the methods used to collect data from reports, including how many reviewers collected data from each report, whether they worked	Section 2.4 Data extraction, handling of multi-arm studies, and statistical analysis

		independently, any processes for obtaining or confirming data from study investigators, and if applicable, details of automation tools used in the process.	
Data items	10a	List and define all outcomes for which data were sought. Specify whether all results that were compatible with each outcome domain in each study were sought (e.g. for all measures, time points, analyses), and if not, the methods used to decide which results to collect.	Section 2.3 Outcomes and mechanism-phenotype mapping; Section 2.4 Data extraction, handling of multi-arm studies, and statistical analysis; Table 1
	10b	List and define all other variables for which data were sought (e.g. participant and intervention characteristics, funding sources). Describe any assumptions made about any missing or unclear information.	Section 2.4 Data extraction, handling of multi-arm studies, and statistical analysis; Table 2
Study risk of bias assessment	11	Specify the methods used to assess risk of bias in the included studies, including details of the tool(s) used, how many reviewers assessed each study and whether they worked independently, and if applicable, details of automation tools used in the process.	Section 2.5 Risk of bias and certainty of evidence assessment; Figure 3
Effect measures	12	Specify for each outcome the effect measure(s) (e.g. risk ratio, mean difference) used in the synthesis or presentation of results.	Section 2.4 Data extraction, handling of multi-arm studies, and statistical analysis
Synthesis methods	13a	Describe the processes used to decide which studies were eligible for each synthesis (e.g. tabulating the study intervention characteristics and comparing against the planned groups for each synthesis (item #5)).	Sections 2.2-2.4; Table 2
	13b	Describe any methods required to prepare the data for presentation or synthesis, such as handling of missing summary statistics, or data conversions.	Section 2.4 Data extraction, handling of multi-arm studies, and statistical analysis
	13c	Describe any methods used to tabulate or visually display results of individual studies and syntheses.	Section 2.4; Figures 4–7; Supplementary Figures S1–S15; Tables 2–3; Supplementary Tables S2–S3 and S6
	13d	Describe any methods used to synthesize results and provide a rationale for the choice(s). If meta-analysis was performed, describe the model(s), method(s) to identify the presence and extent of statistical heterogeneity, and software package(s) used.	Section 2.4 Data extraction, handling of multi-arm studies, and statistical analysis
	13e	Describe any methods used to explore possible causes of heterogeneity among study results (e.g. subgroup analysis, meta-regression).	Section 2.4; Section 3.6; Supplementary Figure S15; Supplementary Table S6

	13f	Describe any sensitivity analyses conducted to assess robustness of the synthesized results.	Section 2.4; Section 3.8; Supplementary Table S3
Reporting bias assessment	14	Describe any methods used to assess risk of bias due to missing results in a synthesis (arising from reporting biases).	Section 2.4; Section 3.9; Supplementary Figures S12-S14
Certainty assessment	15	Describe any methods used to assess certainty (or confidence) in the body of evidence for an outcome.	Section 2.5 Risk of bias and certainty of evidence assessment; Section 3.9; Supplementary Table S2
Study selection	16a	Describe the results of the search and selection process, from the number of records identified in the search to the number of studies included in the review, ideally using a flow diagram.	Section 3.1 Study selection and characteristics of included studies; Figure 2
	16b	Cite studies that might appear to meet the inclusion criteria, but which were excluded, and explain why they were excluded.	Section 3.1; Figure 2; Supplementary Table S5
Study characteristics	17	Cite each included study and present its characteristics.	Section 3.1 Study selection and characteristics of included studies; Table 2
Risk of bias in studies	18	Present assessments of risk of bias for each included study.	Section 3.2 Risk of bias assessment; Figure 3
Results of individual studies	19	For all outcomes, present, for each study: (a) summary statistics for each group (where appropriate) and (b) an effect estimate and its precision (e.g. confidence/credible interval), ideally using structured tables or plots.	Figures 4–7; Supplementary Figures S1–S15; Tables 2–3; Supplementary Tables S2–S3 and S6
Results of syntheses	20a	For each synthesis, briefly summarise the characteristics and risk of bias among contributing studies.	Sections 3.2–3.6; Figure 3; Table 3
	20b	Present results of all statistical syntheses conducted. If meta-analysis was done, present for each the summary estimate and its precision (e.g. confidence/credible interval) and measures of statistical heterogeneity. If comparing groups, describe the direction of the effect.	Sections 3.3–3.6; Figures 4–7; Supplementary Figures S1–S11 and S15; Table 3; Supplementary Tables S2 and S6
	20c	Present results of all investigations of possible causes of heterogeneity among study results.	Section 3.6; Supplementary Figure S15; Supplementary Table S6
	20d	Present results of all sensitivity analyses conducted to assess the robustness of the synthesized results.	Section 3.8 Sensitivity analyses; Supplementary Table S3

Reporting biases	21	Present assessments of risk of bias due to missing results (arising from reporting biases) for each synthesis assessed.	Section 3.9 Publication bias and certainty of evidence; Supplementary Figures S12-S14
Certainty of evidence	22	Present assessments of certainty (or confidence) in the body of evidence for each outcome assessed.	Section 3.9 Publication bias and certainty of evidence; Supplementary Table S2
DISCUSSION	23a	Provide a general interpretation of the results in the context of other evidence.	
DISCUSSION	23b	Discuss any limitations of the evidence included in the review.	Section 4.6 Strengths and limitations
DISCUSSION	23c	Discuss any limitations of the review processes used.	Section 4.6 Strengths and limitations
DISCUSSION	23d	Discuss implications of the results for practice, policy, and future research.	Sections 4.5 Clinical implications and 4.7 Future research directions

DISCUSSION			
Discussion	23a	Provide a general interpretation of the results in the context of other evidence.	Sections 4.1-4.4
	23b	Discuss any limitations of the evidence included in the review.	Section 4.5 Strengths and limitations
	23c	Discuss any limitations of the review processes used.	Section 4.5 Strengths and limitations
	23d	Discuss implications of the results for practice, policy, and future research.	Sections 4.4 Clinical implications; Section 4.6 Future research directions
OTHER INFORMATION			
Registration and protocol	24a	Provide registration information for the review, including register name and registration number, or state that the review was not registered.	Section 2.1 Protocol and reporting guideline
	24b	Indicate where the review protocol can be accessed, or state that a protocol was not prepared.	Section 2.1 Protocol and reporting guideline
	24c	Describe and explain any amendments to information provided at registration or in the protocol.	Section 2.1 Protocol and reporting guideline
Support	25	Describe sources of financial or non-financial support for the review, and the role of the funders or sponsors in the review.	Funding section
Competing interests	26	Declare any competing interests of review authors.	Conflicts of Interest section
Availability of data, code and	27	Report which of the following are publicly available and where they can be found: template data collection forms; data extracted from included studies; data used for all analyses; analytic code; any other materials used in the review.	Data Availability Statement; Supplementary Materials; analytic code availability statement

other materials			

Supplementary Table S5. Full-text reports excluded after eligibility assessment and reasons for exclusion.

No.	Excluded full-text report	Reason for exclusion	Detailed explanation
1	Yang et al., 2026	No usable outcome data	This oral NMN placebo-controlled study used a counterbalanced crossover design and did not provide extractable parallel-group data. Its outcomes focused on skeletal-muscle inflammatory and mechanistic markers after BFR exercise rather than the prespecified hepatic biochemical, metabolic, blood pressure, or group-specific adverse-event outcomes.
2	Zhao et al., 2022	No usable outcome data	This oral NMN randomized placebo-controlled trial mainly reported sleep quality outcomes, including PSQI and smart-band sleep data. It did not provide extractable post-intervention or change data for the prespecified metabolic, blood pressure, hepatic biochemical, or group-specific adverse-event outcomes.
3	Niu et al., 2021	No control group design	The human component was uncontrolled and did not provide an eligible randomized concurrent control group for assessing the independent effect of oral NMN in adults.
4	Yamaguchi et al., 2024	No control group design	This was a single-arm, open-label trial without a randomized concurrent control group.
5	Yamane et al., 2023	No control group design	This was a single-arm before–after study without a placebo or eligible concurrent control group.

6	Kim et al., 2022	No usable outcome data	This oral NMN randomized placebo-controlled trial mainly reported sleep, fatigue, and physical performance outcomes. It did not provide extractable post-intervention or change data for the prespecified metabolic, blood pressure, hepatic biochemical, or group-specific adverse-event outcomes.

Supplementary Table S6. Exploratory subgroup analyses for the effects of NMN supplementation on systolic and diastolic blood pressure

Outcome	Subgroup factor	Subgroup	k	NMN/control, n	MD (95% CI), mmHg	I² (%)	P for subgroup difference
DBP	Age group	Age <60 years	7	108/97	−2.04 (−4.23, 0.16)	0	
DBP	Age group	Age ≥60 years	4	74/60	−3.18 (−6.19, −0.16)	0	0.549
DBP	Metabolic status	Healthy participants	8	139/126	−1.88 (−4.03, 0.28)	0	
DBP	Metabolic status	Metabolic risk	3	43/31	−3.61 (−6.74, −0.47)	0	0.372
DBP	Intervention duration	≥10 weeks	5	85/82	−2.13 (−4.97, 0.70)	0	
DBP	Intervention duration	<10 weeks	6	97/75	−2.63 (−4.90, −0.35)	0	0.79
SBP	Age group	Age <60 years	7	108/97	0.81 (−2.83, 4.44)	53.3	
SBP	Age group	Age ≥60 years	4	74/60	−4.40 (−8.61, −0.20)	0	0.066
SBP	Metabolic status	Healthy participants	8	139/126	0.97 (−2.05, 4.00)	27.1	
SBP	Metabolic status	Metabolic risk	3	43/31	−5.07 (−8.64, −1.51)	0	0.011
SBP	Intervention duration	≥10 weeks	5	85/82	−1.34 (−4.70, 2.01)	0	
SBP	Intervention duration	<10 weeks	6	97/75	−0.36 (−5.54, 4.83)	70.3	0.755

Abbreviations: CI, confidence interval; DBP, diastolic blood pressure; MD, mean difference; NMN, nicotinamide mononucleotide; SBP, systolic blood pressure.

Note: Negative MD values indicate greater reductions in blood pressure in the NMN group compared with the control group. P values for subgroup differences test whether the pooled effect estimates differ across subgroups within each subgroup factor; blank cells indicate that the same subgroup-difference test applies to the preceding subgroup factor.
